# EBV latent membrane protein 1 augments γδ T cell cytotoxicity against nasopharyngeal carcinoma by induction of butyrophilin molecules

**DOI:** 10.7150/thno.78395

**Published:** 2023-01-01

**Authors:** Yue Liu, Ka Sin Lui, Zuodong Ye, Tsz Yan Fung, Luo Chen, Ping Yiu Sit, Chin Yu Leung, Nai Ki Mak, Ka-Leung Wong, Hong Lok Lung, Yoshimasa Tanaka, Allen Ka Loon Cheung

**Affiliations:** 1 Department of Biology, Faculty of Science, Hong Kong Baptist University, Hong Kong SAR, China.; 2 Department of Chemistry, Faculty of Science, Hong Kong Baptist University, Hong Kong SAR, China.; 3 Center for Medical Innovation, Nagasaki University, Nagasaki, Japan.

**Keywords:** Nasopharyngeal carcinoma, γδ T cells, butyrophilin, NLRC5, EBV latent membrane protein 1 (LMP1)

## Abstract

Nasopharyngeal carcinoma (NPC) is a diverse cancer with no well-defined tumor antigen, associated with oncogenic Epstein-Barr Virus (EBV), and with usually late-stage diagnosis and survival <40%. Current radiotherapy and chemotherapy have low effectiveness and cause adverse effects, which calls for the need of new therapy. In this regard, adoptive immunotherapy using γδ T cells has potential, but needs to be coupled with butyrophilin 2A1 and 3A1 protein expression to achieve tumoricidal effect.

**Methods:** Human γδ T cells were expanded (with Zol or PTA) and used for cytotoxicity assay against NPC cells, which were treated with the EBV EBNA1-targeting peptide (L_2_)P_4_. Effect of (L_2_)P_4_ on BTN2A1/BTN3A1 expression in NPC cells was examined by flow cytometry and Western blot. An NPC-bearing NSG mice model was established to test the effectiveness of P_4_ and adoptive γδ T cells. Immunofluorescence was performed on NPC tissue sections to examine the presence of γδ T cells and expression of BTN2A1 and BTN3A1. EBV gene expression post-(L_2_)P_4_ treatment was assessed by qRT-PCR, and the relationship of LMP1, NLRC5 and BTN2A1/BTN3A1 was examined by transfection, reporter assay, Western blot, and inhibition experiments.

**Results:** Zol- or PTA-expanded the Vδ2 subset of γδ T cells that exerted killing against certain NPC cells. (L_2_)P_4_ reactivates latent EBV, which increased BTN2A1 and BTN3A1 expression and conferred higher susceptibility towards Vδ2 T cells cytotoxicity *in vitro*, as well as enhanced tumor regression *in vivo* by adoptive transfer of Vδ2 T cells. Mechanistically, (L_2_)P_4_ induced EBV LMP1, leading to IFN-γ/p-JNK and NLRC5 activation, and subsequently stimulated the expression of BTN2A1 and BTN3A1.

**Conclusions:** This study demonstrated the effectiveness of using the EBV-targeting probe (L_2_)P_4_ and adoptive γδ T cells as a promising combinatorial immunotherapy against NPC. The identification of the LMP1-IFN-γ/p-JNK-NLRC5-BTN2A1/BTN3A1 axis may lead to new insight and therapeutic targets against NPC and other EBV^+^ tumors.

## Introduction

Nasopharyngeal carcinoma (NPC) is a malignancy of the nasopharynx tissues. It is more common found in southern China and Southeast Asian regions that represents 80% of globally new cases 1. The exact cause of NPC remains to be defined but it has been associated with alcohol intake, diet, and smoking 2. However, symptoms are not apparent until late stages of NPC, where combinations of chemotherapy, radiotherapy (e.g. intensity-modulated radiotherapy) and surgery are unable to rectify the tumor 3-7. Alternative therapy such as adoptive immunotherapy was developed in recent years for NPC patients, but the effectiveness of transferred cells is dampened due to the immunosuppressive tumor microenvironment (TME) 8. Moreover, the association of the Epstein-Barr Virus (EBV) in the development of NPC worsens the matter. Recent genomic studies have defined the relationship between Epstein-Barr Virus (EBV) latent genes and genetic mutation of MAPK/PI3K, HLA class I, and aberrant NF-κB that govern the onset and progression of NPC 9, 10.

EBV is a ubiquitous gammaherpesvirus that can undergo lifelong latent infection in the human host. The virus initially infects both B cells and oropharyngeal epithelial cells, and eventually establishes latency and express a limited subset of viral genes known as latent genes 11. In B cells, they usually express type III latent genes such as non-coding RNAs (EBERs), nuclear proteins (EBNA-LA, EBNA1, EBNA2), and membrane proteins (LMP1, LMP2A) 12. On the other hand, EBV can undergo latency in nasopharynx epithelium, where type II latency genes are expressed, such as oncogenic EBNA1, LMP1, LMP2A and EBERs 13. These EBV-encoded latent genes can interact with host oncogenes resulting in disturbed host cell cycle and promoting the development of EBV-associated epithelial neoplasms. For example, latent membrane protein 1 (LMP1) is a transmembrane protein that structurally contains a short N-terminal cytoplasmic domain, six transmembrane loops, and a long cytoplasmic tail consisting of three C-terminal activation regions (CTAR) in the positional order of CTAR -1, -3 and -2 14. Functionally, CTAR-1 and CTAR-2 domains of LMP1 can activate a number of signaling cascades, including the PI3K-AKT, NF-κB, MAPK, JNK, JAK-STAT and p38/MAPK pathways 15, which confers to tumor transformation, immune suppression and inflammatory responses. There are fewer functional studies on CTAR3 where it was shown to bind JAK3 to facilitate DNA binding of STAT; or it binds Ubc9 to trigger migration of tumor cells 16, 17. Thus, EBV maintains latency by modulating the cellular environment that could result in malignant nasopharyngeal epithelial cells 18. Attempts have been made to reactivate EBV for oncolytic activities but the subsequent collateral cell damage, immunological cell death (ICD), and inflammation may allow residual cancer cells to grow 14. More recently, a peptide inhibitor (L_2_P_4_ or P_4_) that blocks EBNA1 dimerization in the nuclei of NPC cells was shown to inhibit tumor growth, which may have implications in assisting immune cell mediated killing 19.

The Vδ2 subset of human γδ T cells has been used as immunotherapeutic cells against different types of cancer. Clinical trials have shown, to a certain extent, the effectiveness of these cells in limiting tumor growth especially in hematological malignancies but less so in solid tumors 20-22. Although Vδ2 T cells consists of 1-5% of peripheral blood lymphocytes, they can be expanded robustly using zoledronic acid (Zol) or tetrakis-pivaloxloxymethyl 2-(thiazole-2-ylamino) ethylidene-1,1-bisphosphonate (PTA) for anti-tumor cytotoxic activities 20, 23-25. Vδ2 T cells are activated by recognition of phosphoantigens (pAgs) presented by BTN2A1/BTN3A1 on the tumor cells 26-28. However, the expression of BTN2A1 and BTN3A1 on NPC cells, and whether Vδ2 T cells are effective against EBV-bearing NPC, have not been characterized to date. We, therefore, hypothesized that Vδ2 T cells has potent cytotoxic activity against NPC when BTN2A1/BTN3A1 expression are increased.

In this study, we investigated the effectiveness of expanded Vδ2 T cells against NPC in combination of the EBV-reactivating agent (L_2_)P_4_ and the potential mechanism in *in vitro* and *in vivo* experiments. First, we showed that there is a differential Vδ2 T cell killing activity against NPC cell lines that is related to the expression of BTN2A1 and BTN3A1. Second, (L_2_)P_4_ increased the protein expression of BTN2A1/BTN3A1 in NPC cells and conferred greater susceptibility towards Vδ2 T cell cytotoxicity. Third, (L_2_)P_4_ induced EBV LMP1 gene stimulates IFN-γ and JNK pathway to upregulate the expression of BTN2A1/BTN3A1 via NLRC5 (NOD-like receptor family CARD domain containing 5). Finally, using an immunodeficient mice model to bear NPC tumors, adoptive transfer of Vδ2 T cells combined with P_4_ led to tumor regression. Taken together, these findings present a potentially effective combinatorial immunotherapy by using a EBV reactivation stimulant and Vδ2 T cell adoptive transfer.

## Materials and Methods

### Cells

HK1, HK1-EBV, HONE1, HONE1-EBV cells were maintained with Dulbecco's modified Eagle's medium (DMEM; GIBCO) supplemented with 10% fetal bovine serum (FBS; GIBCO) and 1% Pencillin/Streptomycin (GIBCO). C666-1 and NPC43 cells were maintained in Roswell Park Memorial Institute (RPMI)1640 medium supplemented with 10% FBS and 1% Pencillin/Streptomycin. 4 μM ROCK inhibitor Y27632 (Cat. No. S1049, Selleckchem) was added to NPC43 culture medium. Gastric tumor cells SNU719 and NCC224 (Korean Cell Line Bank) were cultured in RPMI1640 supplemented with 10% FBS. AGS (Korean Cell Line Bank) were cultured in Ham's F-12K (Kaighn's) Medium (GIBCO) supplemented with 10% FBS. Peripheral blood mononuclear cells (PBMCs) were isolated from healthy human buffy coats (Hong Kong Red Cross) using Lymphoprep (Stem Cell Technologies) with the approval of the institutional review board. All cells were maintained at 37°C and 5% CO_2_ incubator.

### *In vitro* expansion and purification of γδ T cells

PBMCs (2 x 10^6^/well/ml) were stimulated with zoledronic acid (Zol, 1 μM; Cat. No. 1724827, United States Pharmacopeia (USP)) or tetrakis-pivaloxloxymethyl 2-(thiazole-2-ylamino) ethylidene-1,1-bisphosphonate (PTA, 1 μM) 25, in RPMI1640 medium with recombinant human interleukin-2 (IL-2 or rhIL-2; Cat. No. 130-097-746, Miltenyi Biotec) at 100 IU/ml and 10% FBS. Negative selection using magnetic beads in TCRγ/δ^+^ T Cell Isolation Kit (Cat. No. 130-092-892, Miltenyl Biotec) was performed on day 9 of expansion to achieve a purity of >95% CD3^+^Vδ2^+^ determined by flow cytometry before used for experiments.

### Reactivation of EBV in NPC cells

To induce EBV reactivation in NPC cells, sodium butyrate (NaB, 3 mM; Sigma) and phorbol 12-myristate 13-acetate (PMA, 40 ng/ml; Sigma), L_2_P_4_ or P_4_ (10 μM) were added to the culture medium and incubated at 37 °C for 24 and 48 h 19. NPC cells were seeded overnight before treatment occurred. Reactivation was confirmed by monitoring EBV DNA copy number by qPCR and viral gene expression by RT-qPCR (see below).

### Inhibitor experiment

Inhibitors were used prior to P_4_ treatment in NPC cells: JSH-23 (10 μM; Cat. No. HY-13982, MedChemExpress) against NF-κB, SB 203580 (50 nM; Cat. No. HY-10256, MedChemExpress) against p38, JNK inhibitor VIII (50 nM/ 160 nM; Cat. No. HY-10526, MedChemExpress) against JNK1 and JNK2 respectively, or DMSO control (Cat. No. D8418, Sigma). The cells were incubated at 37°C for 1 h, followed by P_4_ (10 μM) treatment and were further incubated at 37°C for 24 h. Cells were seeded overnight to reach 70-80% confluence before treatment occurred.

### Gene-silencing experiments

Small interfering RNA (siRNA) duplexes targeting NLRC5 expression (siNLRC5_1 and siNLRC5_2) were purchased from GenePharma and were used to transfect HONE1-EBV cells using Lipofectamine 3000 (Life Technologies) according to the manufacturer's instructions. The sequences of the oligonucleotides for NLRC5 used are as follows: siCtrl, 5'-UUCUCCGAACGIGUCACGU-3'; siNLRC5_1, 5'-CAGGGUUCUCUCCCUGUUAGA-3'; and siNLRC5_2, 5'-GCUGAUCUUUGAUGGGCUA-3'. 24 h post-transfection, the cells were treated with P_4_ for another 16-24 h where the cells were analyzed for NLRC5, BTN3A1 and BTN2A1 protein expression by Western blotting. For the knockdown of BTN2A1, three duplexes of siBTN2A1 were purchased from Origene and used in C666-1 cells and decreased protein expression was verified by Western blotting. C666-1 cells pre-treated with siBTN2A1 for 24 h before treated with or without P_4_ for 12 h and then co-cultured with Vδ2 T cells at E:T=10:1 ratio for cytotoxicity assay using the same procedures as described below.

### Flow cytometry analysis

2 x 10^5^ cells collected from the cell culture were washed in phosphate buffered saline (PBS) (+1% FBS) and stained with antibodies for 30 minutes at 4°C before analyzed on the BD FACS Caliber II instrument. Antibodies used are shown in **Table S1**.

### Cytotoxicity assay

1 x 10^6^ NPC cells per ml of PBS were labeled with 0.8 mM Calcein-AM (Invitrogen) according to manufacturer's instructions. Co-culture with purified γδ T cells at different (E:T) ratios in triplicates occurred in a U-bottom cell culture plate (Thermo Fisher Scientific), and incubated for 4 hours in a 37°C and 5% CO_2_ incubator. The plate was centrifuged for 5 min at 400 xg before the supernatant was transferred to a black 96-well plate to measure released fluorescence signals at 495 nm excitation and 515 nm emission wavelengths. Cell lysis percentage was calculated as 100 x [(experimental release - spontaneous release) ÷ (maximum release - spontaneous release)]. Maximum release is determined by lysis of the target cell with a final concentration of 0.1% Triton X-100.

### Western blotting analysis

Cells were lysed and protein concentrations were determined using the Pierce BCA protein assay kit (Thermo Fisher Scientific). Equal amounts of protein lysates were loaded onto 4-12% SDS-PAGE gel for electrophoresis and transferred to the PVDF membrane (Millipore) using Bio-Rad Wet/Tank Blotting System. The membrane blot was blocked for an hour in 5% skimmed milk plus 0.5% Bovine Serum Albumin (BSA; Cat. No. A2153, Sigma) in Tris-buffered saline with 0.1% Tween-20 (TBS-T) on a gentle rocking platform. Primary and secondary HRP antibodies used are shown in **Table S1**. Pierce ECL Western Blotting Substrate was used for chemiluminescence (Cat. No. 32106, Thermo Fisher Scientific) and images taken using ChemiDoc (Bio-Rad). Analysis of band intensities was performed using ImageJ (http://imagej.nih.gov/ij/).

### Reverse transcription quantitative real-time PCR (RT-qPCR)

RT-qPCR was performed using total RNA isolated with RNAiso PLUS (Cat. No. 9109, Takara), followed by cDNA synthesis using the PrimeScript RT Reagent Kit (Cat. No. RR047A, Takara). cDNA with specific primers were used with TB Green Premix Ex Taq II (Ti RNase H Plus) (Cat. No. RR820A, Takara) for qPCR analysis. *GAPDH* gene expression was used for normalization. Gene primer sets used are shown in **Table S2**.

### Clustering and principal component analysis

RT-qPCR data on EBV genes expression and the gene expression of *BTN2A1* and *BTN3A1* over the time course of 1, 2, 4, 8, 12, 24 h post-infection (P.I.) were analyzed using clustering and principal component analysis (PCA). Relative expression of the genes normalized to housekeeping gene *GAPDH* for the individual NPC cell lines were inputted into the online clustering software (Clustergrammar; https://maayanlab.cloud/clustergrammer/) 29. Genes clustered together are indicated by a bracket. PCA were conducted using the same set of data by the PCA analysis component with the standardized method with the “Kaiser rule” in GraphPad Prism version 9.

### Cell transfection

LMP1 (wildtype, and mutants 3C, 8A, 3C+8A), BZLF1 and BRLF1 plasmids were kindly provided by Dr. Anna Chi Man Tsang (The Chinese University of Hong Kong) and Dr. Ming Han Tsai (National Yang-Ming University). NPC cells were seeded overnight before culture medium was changed to Opti-MEM (Cat. No. 31985062, GIBCO) 1 h before transfection. Plasmids were transfected into cells using PEI (Cat. No. 23966-1, Polysciences) prepared in Opti-MEM. PEI alone served as vehicle control. Six hours later, the medium was changed to fresh warm DMEM with 10% FBS. Plasmid encoding NLRC5 (#37509) and IFN-γ promoter luciferase (#17599) were purchased from Addgene.

### EBV genome copy number assay

Cellular DNA was extracted using the Genomic DNA Purification Kit (Cat. No. K0721, Thermo Scientific) according to the manufacturer's instruction. To determine EBV genome copy number, 20 μl qPCR reactions using specific primers for the BZLF1 gene was performed with the TB Green Premix Ex Taq II as described above. The plasmid for the standard curve, which encodes the BZLF1 gene region of the EBV genome (10^2^, 10^3^, 10^4^, 10^5^, 10^6^, 10^7^ and 10^8^ copies per 2 μL), were used to generate a standard curve by qPCR based on the CT values. The concentration of BZLF1 DNA in the test samples was quantified using this standard curve. To determine the number of cells in the input template, primers for CCR5 were performed with a standard plasmid as described previously 30.

### Luciferase reporter assay

Luciferase reporter plasmids were created by inserting the promoter regions of BTN2A1, BTN3A1, IFN-γ and NLRC5 into pGL3 basic plasmid using XhoI-HindlII. Inserts were obtained either by PCR amplification using the Q5® High-Fidelity 2X Master Mix (NEB) or using annealed purchased DNA oligos (IGE bio). The pGL3 plasmid was used as negative control. All constructs were verified by sequencing. For the experiment, HEK293T cells were seeded into a 24-well plate and luciferase reporter plasmids were co-transfected the next day using PEI transfection reagent with plasmids encoding EBV genes. In certain experiments, IFN-γ (Peprotech) were added to the cell culture 24 h post-transfection. An empty backbone was used as control (Ctrl). The pRLTK (Renilla) luciferase reporter was included for normalization. Cells were harvested between 30 and 40 h post-transfection and cell lysates were analyzed using the Dual-Luciferase® Reporter Assay System (Promega) following manufacturer's instruction. Bioluminescence was measured using the GloMAX^TM^ 96 Microplate Luminometer (Promega).

### Animal experiments

Four- to six-week-old *NOD.Cg-Prkdc^scid^Il2rg^tm1Wjl^/SzJ* (NSG) mice (at 18-20 g in weight) were obtained from the City University of Hong Kong Laboratory Animal Research Unit. Male and females were equalized between groups, and mice were chosen randomly. All mice experiments were approved by the Research Ethics Committee of Hong Kong Baptist University, and conformed to all relevant regulatory standards. 5 × 10^6^ HONE1-EBV, HK1-EBV, or C666-1 cells (dislodged by trypsin-EDTA (GIBCO)) were resuspended in 0.1 ml RPMI1640 medium and mixed 1:1 with Matrigel basement membrane matrix on ice (LDEV-free; Cat. No. 356232, Corning) before subcutaneously (s.c.) injected into the right flanks of each mouse. Tumor can be detected by day 7. Intratumoral (i.t.) injection of P_4_ (12 μg/100 μl PBS) occurred for 24 h. Vδ2 T cells were recovered from liquid nitrogen storage, placed in RPMI1640 (10% FBS with 20 IU/ml IL-2) overnight, and passed through a 70-μm cell strainer before adoptively transferred to the mice by intravenous (i.v.) injection at 5 x 10^6^ cells in 100 μl PBS. PBS injection alone served as control. Tumor volume (V) was measured by caliper every 2-4 days and calculated with the formula V = (length × width^2^)/2. Mice were sacrificed after 3-4 weeks post-treatment. Dissected tumors were fixed with 10% formalin (Cat. No. HT501128, Sigma) at room temperature for 24 h before processed for paraffin embedding (Cat. No. P3808, Sigma).

### Immunofluorescence staining on tumor tissue sections

Formalin-fixed paraffin-embedded (FFPE) tissue sections of the tumors from the mice experiments were used for immunofluorescence assays as previous 31. Antibodies used are shown in **Table S1**. Nuclei were stained using Hoechst 33258 Staining Dye Solution (Cat. No. ab228550, Abcam). Images were acquired using the SP5 II or Stellaris confocal microscopy system (Leica) and analyzed using the LAS X (Leica) and ImageJ softwares.

### Hematoxylin and eosin (H&E) staining

Tissue sections (5 μm thick) of the tumors from the mice experiments were stained with hematoxylin (Cat. No. GHS116, Sigma) and eosin (Cat. No. HT110116, Sigma) (H&E) after deparaffinization with xylene (Cat. No. 1330-20-7, RCI Labscan) and rehydration with ethanol. Images were acquired using the ECLIPSE Ti2 inverted microscope (Nikon).

### Statistical analysis

Data shown are the mean ± SEM from at least three independent experiments. Statistical analyses were performed using paired Student's *t-test*, one-way or two-way analysis of variance (ANOVA), unless otherwise indicated. *P* < 0.05 is considered statistically significant.

## Results

### NPC cells are susceptible to γδ T cell killing

**Figure 1:**
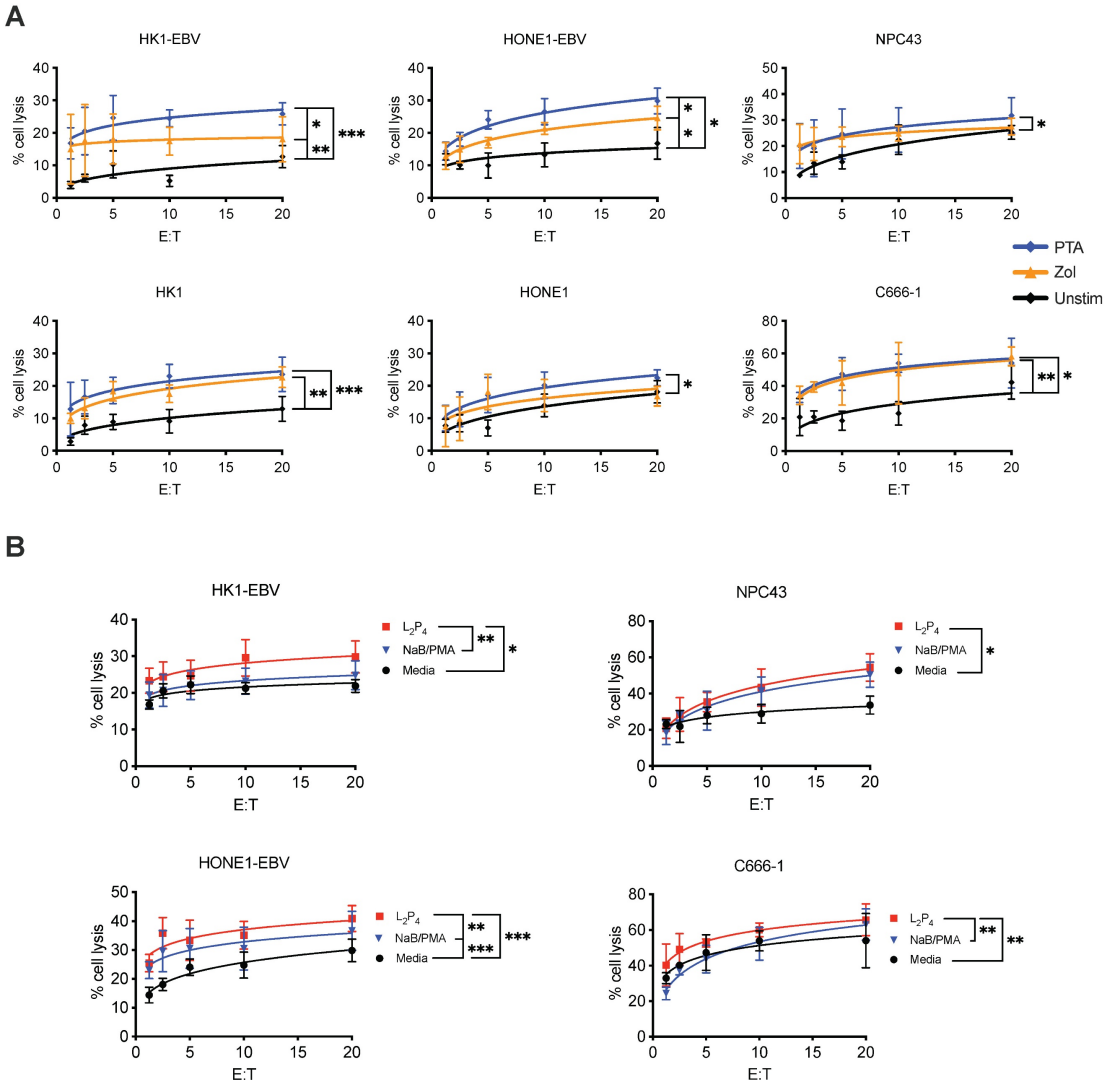
** Vδ2 T cell cytotoxicity towards NPC cell lines. (A)** NPC cell lines (HONE1-EBV, HK1-EBV, C666-1, NPC43) were used for Calcein cytotoxicity assay after co-culture with PTA- or Zol- expanded Vδ2 T cells at different effector: target (E:T) ratios for 4 h. **(B)** NPC cell lines were pre-treated with NaB/PMA or L_2_P_4_ for 24 h before co-cultured with PTA-expanded Vδ2 T cells. Calcein release at 4 h post co-culture were measured to calculate % cell lysis based on maximum cell death. Data represents mean ± SEM from 5 independent experiments. One-way ANOVA statistical test was performed. *P < 0.05, **P < 0.01, ***P < 0.001.

Cytotoxicity assay was performed to determine if γδ T cells can kill NPC cells. Expansion of Vδ2 T cells was achieved by stimulating healthy donor PBMCs with PTA or Zol plus IL-2 for 9 days, where cell clusters can be detected under light microscopy (**Figure S1A**). By the end of culture, up to 40-fold increase in the number of CD3^+^Vδ2^+^ T cells was found with a purity of >90% for both Zol or PTA stimulation (**Figure S1 B and C**). Cells were further purified by negative selection microbeads to ensure a purity of >95% CD3^+^Vδ2^+^ cells (hereafter referred to as Vδ2 T cells) for further experiments. These cells were then co-cultured at different effector: target (E:T) ratios with various NPC cell lines - HK1-EBV, HK1, HONE1-EBV, HONE1, NPC43, and C666-1. HONE1-EBV and HK1-EBV are cell lines latently infected with lab-adapted EBV, whereas C666-1 and NPC43 are clinically relevant cell lines with natural latent EBV 32. Different degree of cytotoxicity by Vδ2 T cells was observed in the order of C666-1> HONE1-EBV=NPC43> HK1-EBV> HK1=HONE1, and were significantly improved when compared to unstimulated CD3^+^Vδ2^+^ T cells (**Figure 1A**). Moreover, PTA-expanded Vδ2 T cells (PTA-Vδ2) had significantly increased level of cytotoxicity than Zol-Vδ2 against HK1-EBV and HONE1-EBV cells (**Figure 1A**). These results suggest that EBV-bearing NPC cell lines appear to be more susceptible towards Vδ2 T cell killing.

### Reactivation of EBV in NPC cells increased Vδ2 T cell killing

To understand the role of EBV in the NPC cells that contributes to increased Vδ2 T cell cytotoxicity, we treated HK1-EBV, HONE1-EBV, NPC43 and C666-1 overnight with a EBNA1-targeting peptide inhibitor in a fluorescent-labeled form (L_2_P_4_) or unlabeled form (P_4_) 33, or sodium butyrate/phorbol 12-myristate 13-acetate (NaB/PMA) 34, 35, prior to cytotoxicity experiments. P_4_ can induce EBV reactivation as measured by qPCR for viral DNA copy number (**Figure S2**) and gene expression (discussed below in **Figure S10**). As shown in **Figure 1B**, L_2_P_4_ significantly increased the Vδ2 T cell killing of the treated NPC cell lines by up to 3-fold, especially for those that were found to be more resistant to Vδ2 T cells in the data above. L_2_P_4_ was more effective than NaB/PMA in enhancing Vδ2 T cell cytotoxicity for HK1-EBV, HONE1-EBV and C666-1, but was similar for NPC43 (**Figure 1B**). The effect was not due to the L_2_P_4_ induced cell death that remained minimal in these experiments (**Figure S3**). No significant difference was observed for cytotoxicity against EBV-negative HK1 and HONE1 cells (**Figure S4A**). P_4_ treatment for EBV-positive gastric tumor cells SNU719 and NCC224 still had an improved cytotoxic effect for PTA-Vδ2 cells but not for the EBV-negative AGS cells (**Figure S4B**).

### Adoptive transfer of Vδ2 T cells reduced P_4_-treated NPC tumor growth *in vivo*

**Figure 2:**
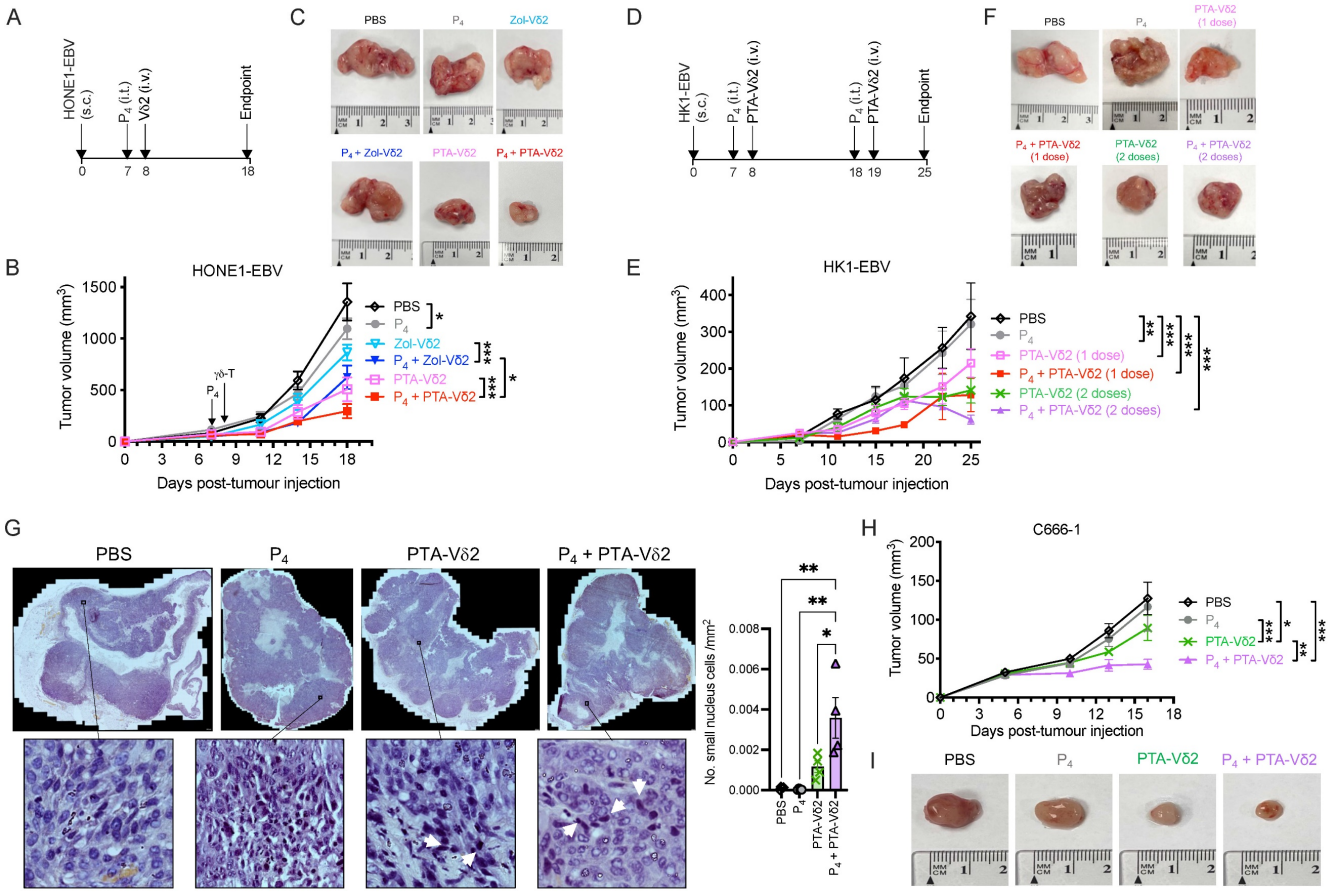
** P_4_ + Vδ2 T cells inhibited NPC tumor growth *in vivo*.** (**A**) Timeline of HONE1-EBV-bearing NSG mice experiment. (**B**) Tumor volume of mice receiving PBS (n = 9), P_4_ (n = 9), Zol-Vδ2 (n = 5), P_4_ + Zol-Vδ2 (n = 5), PTA-Vδ2 (n = 9), and P_4_ + PTA-Vδ2 (n = 9) over a period of 18 days. (**C**) Representative HONE1-EBV tumors from each group at experimental endpoint. (**D**) Timeline of HK1-EBV-bearing NSG mice experiment. (**E**) Tumor volume of mice receiving PBS (n = 4), P_4_ (2 doses, n = 4), PTA-Vδ2 (1 dose, n = 4), P_4_ + PTA-Vδ2 (1 dose, n = 4), PTA-Vδ2 (2 doses, n = 4), and P_4_ + PTA-Vδ2 (2 doses, n = 4) over a period of 25 days. (**F**) Representative HK1-EBV tumors from each group at experimental endpoint. (**G**) Tile scan of HK1-EBV H&E tumor sections with insets to show infiltration of small nuclei cells among the different groups receiving two doses of treatment. Counts per mm^2^ is shown as column graph. (**H**) Tumor volume of C666-1-bearing mice receiving two-dose treatments of PBS (n = 5), P_4_ (n = 5), PTA-Vδ2 (n = 5), and P_4_ + PTA-Vδ2 (n = 5), with representative tumor photos (**I**). Data represents mean ± SEM. Two-way ANOVA statistical test was used for B and E, Student's *t-*test was used for G. *P < 0.05, **P < 0.01, ***P < 0.001.

Next, we sought to test whether Vδ2 T cells combined with (L_2_)P_4_ can effectively target NPC tumors *in vivo*. We used the immunodeficient *NOD*.Cg-*PrkdcscidIl2rgtm1Wjl/SzJ* (NSG) mice to establish an NPC tumor mice model. Subcutaneous (s.c.) injection of HONE1-EBV resulted in the formation of solid tumors by day 7. On the same day, intratumoral (i.t.) injection of P_4_ or PBS was performed 33. 24 h later, Zol- or PTA-Vδ2 cells were adoptively transferred by intravenous (i.v.) injection (**Figure 2A**). PBS injections served as control. For HONE1-EBV, decreased tumor growth by ~40% was achieved for Zol-Vδ2 cells and ~55% for PTA-Vδ2 cells compared to control (**Figure 2 B** and** C**). Interestingly, combining P_4_ treatment with Vδ2 T cell adoptive transfer led to further reduction in tumor size by an average of 52% for Zol-Vδ2 cells and 73% for PTA-Vδ2 cells with statistical significance, respectively (**Figure 2 B and C**). P_4_ alone reduced tumor size modestly by ~19% at the endpoint. Individual tumor photos can be found in **Figure S5A**. Consistent to the *in vitro* data, PTA-Vδ2 cells had a higher anti-NPC activity in these mice experiments compared to Zol-Vδ2 cells, especially when combined with P_4_.

In another set of experiment, two doses of P_4_ plus PTA-Vδ2 treatment were attempted. HK1-EBV tumors detectable by day 7 were (i.t.) injected with P_4_ followed by PTA-Vδ2 cells adoptive transfer on day 8. The second treatment occurred on day 18 (P_4_) and day 19 (PTA-Vδ2 cells) (**Figure 2D**). PTA-Vδ2 cells alone reduced tumor growth by ~54%, while P_4_ plus PTA-Vδ2 cells decreased tumor growth by ~63% compared to control (**Figure 2 E** and** F**). Two doses of PTA-Vδ2 cells or P_4_ plus PTA-Vδ2 cells resulted in a further 8% and 17% tumor size reduction, respectively (**Figure 2 E** and** F, Figure S5B**).

**Figure 3:**
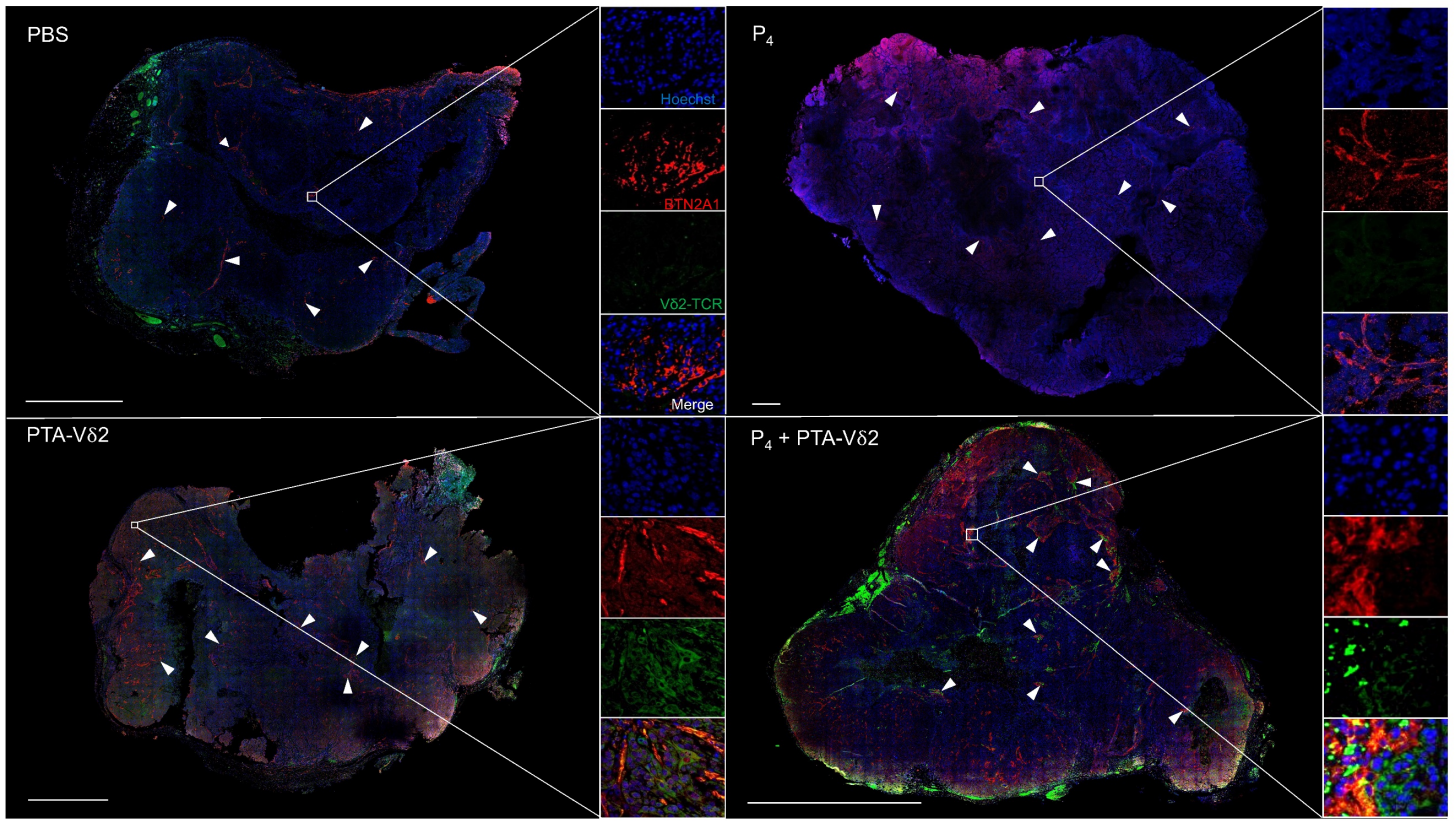
** P_4_ treatment results in increased co-localization of V**δ**2 T cells and BTN2A1-expressing cells in NPC tumor *in vivo*.** HK1-EBV tumor tissue sections from PBS, P_4_, PTA-Vδ2, and P_4_ + PTA-Vδ2 mice were immunostained for Vδ2-TCR+ cells (green), BTN2A1 (red) and nucleus (blue) with Hoechst 33258. Scale bar represents 800 μm. Representative tile scan images assessed by confocal microscopy are shown. Insets are expanded views of the squared regions. Arrows point to regions of BTN2A1 expressing cells.

To examine if P_4_ leads to increased Vδ2 T cell infiltration into the NPC tumor, H&E staining of the HK1-EBV tumor tissue sections was performed. Cells with smaller nuclei (likely Vδ2 T cells) can be found within the HK1-EBV tumors in mice that received PTA-Vδ2 cells (**Figure 2G**). P_4_ injection resulted in the formation of lesions in certain regions of the tile scanned tumor structure where increased number of small-nucleated cells per mm^2^ could be observed following administration of PTA-Vδ2 cells (**Figure 2G,** *white arrows*). Consistently, the presence of Vδ2-TCR^+^ cells (green) in the tumors were observed from mice with adoptively transferred PTA-Vδ2 cells, which seems to have increased in P_4_-treated tumors and were found in close proximity to regions with BTN2A1-expressing cells (**Figure 3,** *red signals and indicated by white arrows*, and **Figure S6**), which are relatively more abundant in the areas surrounding the lesion (**Figure S6**). In comparison, non-P_4_ treated tumor had considerably less BTN2A1 expression and Vδ2-TCR^+^ cells appear to be more randomly distributed in the tumor (**Figure 3**). Since two doses of P_4_ + PTA-Vδ2 cells appear to be more effective, we next tested this in the more clinically relevant C666-1 tumor bearing mice. Compared to two doses of P_4_ or PTA-Vδ2 cells, P_4_ + PTA-Vδ2 cells resulted in significantly decreased tumor size (**Figure 2 H and I**). No significant weight loss or death was found for any of the NPC tumor-bearing mice in the treatment groups prior to ethical sacrifice because of tumor size (**Figure S7**). Taken together, the *in vivo* experiments demonstrated that P_4_ combined with adoptive transferred PTA-Vδ2 cells can successfully reduce NPC tumor growth likely due to the increased cytotoxic activity of the tumor-infiltrated PTA-Vδ2 cells to the BTN2A1-expressing cells.

### P_4_ upregulates expression of BTN3A1 and BTN2A1 on NPC cells

**Figure 4:**
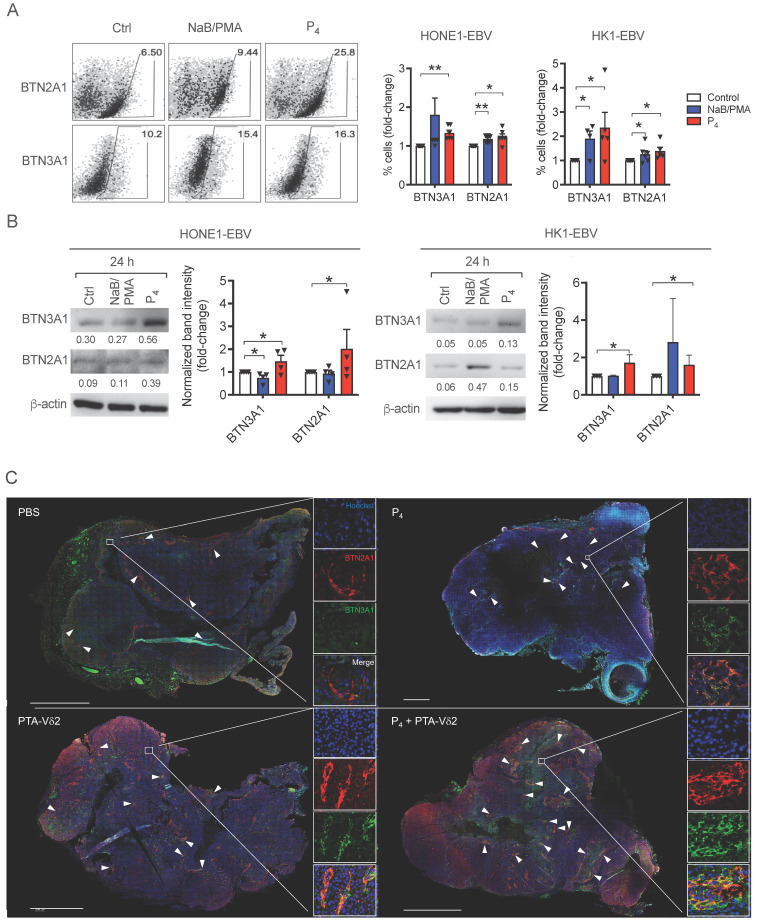
(**A-B**) P4 treatment results induced increased protein expression of BTN2A1 and BTN3A1 co- expression in EBV^+^ NPC cells *in vitro* and *in vivo*. Analysis of BTN3A1 and BTN2A1 expression induced by NaB/PMA or P_4_ 24 h treatment of HONE1-EBV and HK1-EBV cells and normalized to control based on: (A) flow cytometric analysis of frequencies of cells (%) normalized to control. Representative dot plots are shown on the left; and (B) Western blot analysis of band intensities compared to β-actin (shown as numbers). Representative immunoblots are shown. Column graphs represent data as mean ± SEM from ≥ 3 independent experiments. Student's *t-*test was performed. *P < 0.05, **P < 0.01, ***P < 0.001. (**C**) HK1- EBV tumor tissue sections from PBS, P_4_, PTA-Vδ2, and P_4_ + PTA-Vδ2 mice were immunostained for BTN3A1 (green), BTN2A1 (red) and nucleus (blue) with Hoechst 33258. Scale bar represents 800 μm. Representative tile scan images assessed by confocal microscopy are shown. Insets are expanded views of the squared regions. Arrows indicate regions of BTN2A1 expression.

To understand if the increased tumoricidal activity of P_4_ plus PTA-Vδ2 cells is due to increased BTN2A1/BTN3A1 expression, we next analyzed their protein expression on various NPC cells. As shown in **Figure S8**, the expression of both proteins was consistently high in C666-1 and NPC43 cells compared to HK1-EBV, HONE1-EBV, HK1 and HONE1, which in part, correlate with the differences in the *in vitro* cytotoxicity results above (**Figure 1A**). By flow cytometry, 24 h of P_4_ treatment resulted in a consistent increase in the frequency of HONE1-EBV and HK1-EBV cells expressing BTN2A1 and BTN3A1 (**Figure 4A**), which was verified by Western blot analysis (**Figure 4B**). Moreover, we observed an increased expression and co-localization of BTN3A1 and BTN2A1 in P_4_-treated mice tumor tissue sections (i.e. from P_4_ and P_4_ + PTA-Vδ2 group) compared to the non-P_4_-treated mice tumors (i.e. from PBS and PTA-Vδ2 group) (**Figure 4C** and **Figure S9**). Overall, (L_2_)P_4_ stimulated expression of BTN2A1 and BTN3A1, which increased susceptibility for NPC cells towards Vδ2 T cell cytotoxicity. However, how P_4_ induced the expression of BTN3A1 and BTN2A1 in NPC tumors has not been shown to date.

### EBV-encoded LMP1 induces BTN2A1 and BTN3A1 expression via NLRC5

To identify the EBV genes that may be involved in the induction of BTN2A1 and BTN3A1 expression, we first induced EBV reactivation using L_2_P_4_ treatment and traced the gene expression of *BTN2A1* and *BTN3A1*, as well as EBV genes from different classes by RT-qPCR for 1, 2, 4, 6, 12, 24 h post-L_2_P_4_ treatment. Latent EBV gene *LMP1*; immediate-early genes *BRLF1* and* BZLF1*; early genes *BXLF1, BMRF1, BRRF1* and* BARF1*; and late gene *BCRF1* were chosen for analysis due their possibility to modulate host cell immunological responses that may relate to *BTN2A1* and *BTN3A1* expression (**Table S3**). By cluster analysis, the expression profile of *BTN2A1* and *BTN3A1* were grouped with *LMP1* from HONE1-EBV, HK1-EBV and C666-1 and NPC43 cells over time (**Figure S10A**). Principal component analysis (PCA) of the data from the four cell lines showed that *LMP1*, *BZLF1* and *BRLF1* are in the closest distance to *BTN2A1* and *BTN3A1* (**Figure S10B**), suggesting their relationship to *BTN2A1* and *BTN3A1* expression.

**Figure 5:**
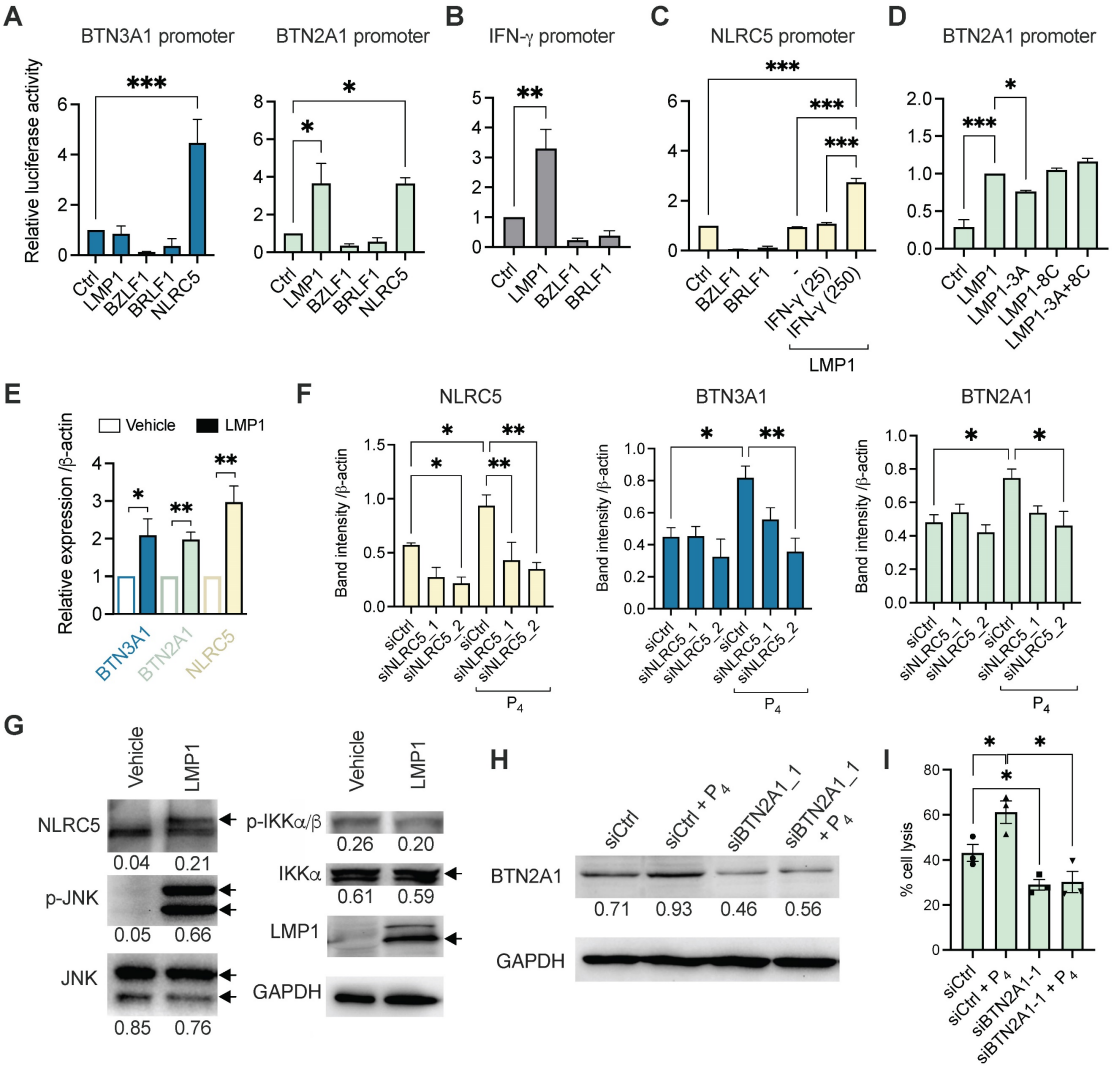
** Mechanistic study of the P_4_-induced LMP1 in stimulating BTN3A1 and BTN2A1 expression via NLRC5.** Luciferase reporter assay using the promoters of (**A**) BTN3A1, BTN2A1, (**B**) IFN-γ and (**C**) NLRC5. HONE1 cells were co-transfected with plasmids encoding *LMP1*, *BZLF1*, *BRLF1* and *NLRC5* for 1 day before results assessed. For **C**, IFN-γ (25 or 250 IU/ml) were used in conjunction. (**D**) Reporter assay using *LMP1*, *LMP1-3A*, *LMP1-8C* or *LMP1-3A+8C* encoded plasmids for the BTN2A1 promoter. (**E**) HONE1 cells were transfected with LMP1 and assessed for *BTN3A1, BTN2A1* and* NLRC5* gene expression by qRT-PCR. (**F**) Two siRNA against NLRC5 or scrambled control were transfected into HONE1-EBV cells for 24 h before treated with P_4_ for another 16 h. Protein expression of NLRC5, BTN3A1 and BTN2A1 were assessed by Western blot and the band intensity compared to control are shown in the column graph. (**G**) Plasmids encoding LMP1 were transfected in HONE1 cells for 5 h before analyzed for NLRC5, p-JNK, JNK, p-IKKα/β and IKKα protein expression by Western blot. Arrows indicate the correct sized band/s. (**H**) Western blot analysis of BTN2A1 expression following transfection of C666-1 cells by siRNA against BTN2A1 with or without P_4_ treatment. These cells were used for cytotoxicity assay co-cultured with Vδ2 cells at 10:1 E:T ratio (**I**). Numbers under immunoblots indicate relative band intensity to GAPDH. Data from 3 independent experiments are shown as mean ± SEM for the column graphs. One-way ANOVA or Student's *t* statistical test was performed. *P < 0.05, **P < 0.01, ***P < 0.001.

To determine if the viral genes can directly trigger the transcription of *BTN2A1* and *BTN3A1* genes, we performed a luciferase reporter experiment using their specific promoters. Previous study demonstrated that BTN3A1 expression can be induced by the immune regulator NLRC5 through direct binding to its promoter 36, and this was included as a control. As shown in **Figure 5A**, BZLF1 and BRLF1 did not have any induction effect on luciferase activity of the BTN2A1 and BTN3A1 promoters. However, LMP1 appears to trigger BTN2A1 promoter transcription but not for BTN3A1. Moreover, NLRC5 could induce BTN3A1 as well as BTN2A1 promoter activities (**Figure 5A**), where the latter has not been shown before. Since LMP1 exists as a membrane-bound protein, we suspect that there is an indirect effect to induce NLRC5 to promote BTN2A1 and BTN3A1 expression, such as IFN-γ. Indeed, LMP1 (but not BZLF1 and BRLF1) can activate the IFN-γ promoter (**Figure 5B**), and when combined with IFN-γ (250 IU/ml) the NLRC5 promoter can become activated (**Figure 5C**). Additionally, overexpressed LMP1 in HONE1 cells or P_4_ treatment of HONE1-EBV cells can induce the gene expression of IFN-γ (**Figure S11**).

To further examine how LMP1 stimulates BTN3A1 and BTN2A1 expression, we performed the luciferase reporter experiments using plasmids encoding mutant LMP1 for the functional domains CTAR1 (3A) and CTAR2 (8C) or both (3A+8C). As shown in **Figure 5D**, only LMP1-3A had a modest but significantly reduced the induction of BTN2A1 promoter activity, suggesting that CTAR1 and downstream pathways may be associated with LMP1 function on regulating BTN2A1 expression. To corroborate the findings, we overexpressed LMP1 in HONE1 cells and found that *BTN3A1*, *BTN2A1* and *NLRC5* gene expression were significantly upregulated by 3 h post-transfection when compared to vehicle (**Figure 5E**). Further, siRNA was used to confirm if NLRC5 mediated BTN3A1 and BTN2A1 expression following P_4_ treatment. HONE1-EBV cells were transfected with two siRNAs against NLRC5 (siNLRC5_1 and siNLRC5_2) for 24 h prior to P_4_ stimulation for another 16-24 h. P_4_ treatment significantly increased NLRC5 protein expression but decreased when either siRNAs against NLRC5 were used (**Figure 5F** and **Figure S12**). P_4_-induced BTN3A1 and BTN2A1 protein levels were also decreased but statistical significance was only reached with siNLRC5_2 (**Figure 5F**). Importantly, P_4_ treatment induced NLRC5 and BTN3A1 co-expression in tumor tissue in our mice experiments compared to those without P_4_ treatment (**Figure S13 A and B**).

LMP1 can activate c-Jun N-terminal kinase (JNK) and NF-κB signaling cascades, we next sought to determine if LMP1 has any effect on these proteins by Western blotting. Overexpression of LMP1 in HONE1 cells after 24 h lead to increased NLRC5 and p-JNK protein levels, suggesting that the NLRC5 pathway was activated (**Figure 5G**). However, p-IKKα/β or IKKα/β had no significant difference, indicating that NF-κB is not induced by LMP1 in HONE1 or it could be inhibited by NLRC5 as reported previously 37. From the results so far, P_4_ treatment of EBV^+^ NPC cells leads to the expression of LMP1, which in turn triggered the expression of IFN-γ and p-JNK to drive NLRC5 induction of BTN3A1 and BTN2A1. Inhibitors against NF-κB and JNK1 slightly decreased the protein expression level of P_4_-induced BTN3A1, BTN2A1, and NLRC5 (**Figure S14**). While inhibition of JNK1 is consistent to the LMP1 transfection results, inhibition of NF-κB led to decreased expression is unexpected. Besides LMP1, other factors arising from P_4_ treatment could trigger IFN-γ more potently (**Figure S11B**) and activate the NF-κB-NLRC5 pathway to induce the expression of BTN3A1 and BTN2A1.

To confirm the significance of P_4_-induced BTN2A1 expression in promoting Vδ2 T cell cytotoxicity, we next downregulated BTN2A1 by siRNA (siBTN2A1_1) prior to P_4_ treatment in C666-1 cells, as it has a more dominant role in the activation of Vδ2 T cells 27. BTN2A1 protein expression was found inhibited by siBTN2A1_1 compared to control (siCtrl) with or without P_4_ treatment (**Figure 5H** and **Figure S15**). When these cells were co-cultured with PTA-Vδ2 cells, a significantly decreased level of cytotoxicity was found compared to control siRNA pre-transfected cells (**Figure 5I**). Therefore, P_4_-induced BTN2A1 expression allows greater activation of PTA-Vδ2 cells for cytotoxicity. Overall, the P_4_-LMP1-IFN-γ/p-JNK-BTN2A1/BTN3A1 axis identified by these experiments likely underlie the effectiveness of the P_4_ plus PTA-Vδ2 T cell therapy against NPC.

## Discussion

NPC is a diverse and heterogenous tumor with no defined antigens or therapeutic targets that render difficulty for the development of an effective treatment 38-40. Here, we demonstrated a new anti-NPC therapy to inhibit NPC tumor growth by using the combination of an EBV-targeting peptide (L_2_P_4_ or P_4_) synergized with adoptive transfer of Vδ2 T cells. The underlying mechanism is associated with (L_2_)P_4_-induced EBV LMP1 triggers NLRC5 to promote BTN2A1/BTN3A1 expression on NPC. To our knowledge, this is the first report to show the linkage between LMP1, NLRC5, BTN2A1/BTN3A1 expression and higher susceptibility towards Vδ2 T cell cytotoxicity.

The anti-tumor characteristics of Vδ2 T cells are well established and have been used in clinical trials against various cancers besides NPC 20. Our data shows that Vδ2 T cells have cytotoxic effects on NPC cell lines to different extent (**Figure 1**). Moreover, we confirmed that PTA has an advantage in promoting the cytotoxic function of Vδ2 T cells compared to Zol in these settings. It has also been suggested by other studies that PTA expanded cells acquired antigen presentation, cytokine capabilities and memory phenotype 25, 41, 42. When EBV reactivation was induced by NaB/PMA or (L_2_)P_4_, there was an increase in the cytotoxic function of Vδ2 T cells. This is due to the upregulated expression of BTN2A1/BTN3A1 for pAg recognition by TCR of Vδ2 T cells 43. Indeed, P_4_-treated NPC tumors from the mice experiments confirmed the increased co-expression of BTN2A1/BTN3A1 (**Figure 4C**). Therefore, it is likely that NPC tumor cells with dysregulated metabolic pathways accumulate pAgs (such as IPP), which has been reported in cancers in the liver, lung, or colon 21. The possible consequence is the presentation of pAg by BTN2A1/BTN3A1. However, whether the (L_2_)P_4-_stimulated pAgs are presented on BTN2A1/BTN3A1 and recognized by Vδ2 T cells in the NPCs remain to be examined.

The effectiveness of Vδ2 T cells as adoptive immunotherapy against solid tumors relies on successful tumor infiltration while retaining cytotoxic function in the TME 20. In this regard, our mice experiments showed that adoptive transfer of Vδ2 T cells can infiltrate into the NPC tumors that is enhanced by P_4_ treatment (**Figure 3** and **Figure S6**). Previous study reported oncolytic activity of (L_2_)P_4_ against NPC tumors when injected twice a week for 4 weeks but provided no information on the mechanism related to EBV genes or Vδ2 T cells 19. In our hands, P_4_ led to deformation of the tumor structure with areas void of cells (lesions) where the transferred Vδ2 T cells appear to accumulate more efficiently when compared to non-P_4_ controls (**Figure 2F** and** Figure 3**). The increased P_4_-induced BTN2A1/BTN3A1 expression possibly triggered higher activation of the infiltrated Vδ2 T cells for cytotoxicity that lead to tumor regression as observed. In this sense, it is understandable that the poor effectiveness of adoptive Vδ2 T cells in clinical trials may be associated with naturally low BTN2A1/BTN3A1 expression in solid tumors. On the other hand, whether the immune-inhibitory molecules such as Galectin-9, PD-L1/L2, Tim-3, Lag-3, and CTLA4 are affected by P_4_ treatment warrants investigation. Besides, whether the increased BTN2A1/BTN3A1 activation signals allow Vδ2 T cells to overcome inhibitory cells such as myeloid-derived suppressor cells (MDSCs), tumor-associated macrophages (TAMs) and regulatory T cells (Tregs) in the TME of NPC remain to be investigated 44.

NLRC5 is a key regulator of MHC class I expression and CD8^+^ T cell responses 45, 46, where the reduced expression in cancers correlates with decreased anti-tumor cytotoxicity 47. NLRC5 could be induced following NF-κB activation via innate immune pathway, but NF-κB can also be inhibited by NLRC5 37. Moreover, blocking of JNK decreased BTN2A1/BTN3A1 expression in P_4_ treated cells, which may be related to LMP1-induced JNK activation as shown in our experiments (**Figure 5G**) 48. One recent study reported that NLRC5 also regulates BTN3A1-3 expression 36, which implies that the lowered NLRC5 expression in solid tumors could also result in the loss of Vδ2 T cell cytotoxic functions. In addition, our reporter assay results found that NLRC5 can also trigger BTN2A1 expression which was not shown before. NLRC5 has been suggested as a form of cancer immunotherapy that complements effective immune checkpoint inhibitor treatment 49. As previous work also identified somatic mutations of NLRC5 in 4.8% of EBV-positive NPCs 40, but whether these mutations affect BTN2A1 and BTN3A1 expression remains to be investigated in clinical NPC cancers. The use of P_4_ treatment in EBV^+^ NPCs to upregulate NLRC5 may provide an advantage to enhance anti-tumor immune responses. The relevance of LMP1 to NLRC5 may reinforce the importance of the EBV gene in anti-tumor immunity.

LMP1 is an oncogene that regulates multiple cellular processes for tumor transformation, including activation of fibroblast growth factor receptor 1 (FGFR1) 50, increased aerobic glycolysis through mTORC1 51, and promotion of epithelial-mesenchymal transition (EMT) 52-54. However, LMP1 has not been shown to induce NLRC5-triggered butyrophilins expression related to Vδ2 T cell cytotoxic functions. In our hands, LMP1 CTAR1 appeared to influence BTN2A1 expression likely through IFN-γ and p-JNK mediated NLRC5. However, CTAR1 can activate other signaling cascades that may have the potential to influence the expression of BTN2A1 and BTN3A1 15. Here, not only did we confirm that NLRC5 can stimulate BTN3A1 expression 36, but it also induces BTN2A1 in NPC cells. Moreover, LMP1 can elevate p-JNK and IFN-γ that may lead to the upregulation of NLRC5. We also postulate that LMP1 induced NLRC5 may also upregulate MHC-I for anti-tumor CD8^+^ T cell responses, which should be examined. Therefore, there is potential for LMP1 as a therapeutic tool to stimulate NLRC5 for enhancing anti-tumor T cell responses in the TME.

Overall, this study demonstrated that Vδ2 T cells can successfully inhibit NPC tumor growth when the hurdle of sufficient BTN2A1/BTN3A1 expression are overcame by use of the EBV-reactivating agent (L_2_)P_4_. The mechanism that P_4_-induced LMP1 can trigger the IFN-γ/p-JNK-NLRC5-BTN2A1/BTN3A1 axis has the potential for future development as a combinatorial immunotherapy with adoptive Vδ2 T cells in treating NPCs as well as other tumors.

## Supplementary Material

Supplementary figures and tables.

## Abbreviations

BCRF1: BamHI-C fragment rightward reading frame 1
BSA: bovine serum albumin
BTN: butyrophilin
BZLF1: BamHI Z fragment leftward open reading frame 1
cDNA: complementary DNA
CTAR: C-terminal activation region
CTLA4: cytotoxic T-lymphocyte-associated protein 4
DMEM: Dulbecco's modified Eagle's medium
E:T: effector: target
EBERs: epstein-barr virus encoded small RNAs
EBNA: epstein-barr nuclear antigen
EBV: epstein-barr virus
EMT: epithelial-mesenchymal transition
FBS: fetal bovine serum
FFPE: formalin-fixed paraffin-embedded
FGFR1: fibroblast growth factor receptor 1
HLA: human leukocyte antigen
HRP: horseradish peroxidase
i.t.: intratumoral
i.v.: intravenous
ICD: immunological cell death
IFN: interferon
IKKα: IκB Kinase α
IPP: isopentenyl pyrophosphate
JNK: c-Jun N-terminal kinases
Lag-3: lymphocyte-activation gene 3
LMP1: latent membrane protein 1
MAPK: mitogen-activated protein kinase
MDSC: myeloid-derived suppressor cell
MFI: mean fluorescence intensity
mTORC1: mammalian target of rapamycin complex 1
NaB: sodium butyrate
NF-κB: nuclear factor kappa-light-chain-enhancer of activated B cells
NLRC5: NOD-like receptor family CARD domain containing 5
NPC: nasopharyngeal carcinoma
NSG: *NOD.Cg-Prkdc^scid^Il2rg^tm1Wjl^/SzJ*
P.I.: post-infection
pAg: phosphoantigen
PBMC: peripheral blood mononuclear cell
PBS: phosphate buffered saline
PCA: principal component analysis
PD-L1: programmed death-ligand 1
PD-L2: programmed death-ligand 2
PI3K: phosphoinositide 3-kinase
PMA: phorbol 12-myristate 13-acetate
PPFE: formalin-fixed paraffin-embedded
PTA: tetrakis-pivaloxloxymethyl 2-(thiazole-2-ylamino) ethylidene-1,1-bisphosphonate
PTA-Vδ2: PTA-expanded Vδ2 T cells
rhIL-2 or IL-2: recombinant human interleukin-2
RPMI: Roswell Park Memorial Institute
RT-qPCR: reverse transcription real-time quantitative PCR
s.c.: subcutaneous
siRNA: small interfering RNA
TAM: tumor-associated macrophages
TCR: T-cell receptor
Tim-3: T-cell immunoglobulin and mucin-domain containing-3
TME: tumor microenvironment
TPA: 12-O-tetradecanoylphorbol-13-acetate
Treg: regulatory T cells
V: tumor volume
Zol: zoledronic acid
Zol-Vδ2: zoledronic acid-expanded Vδ2 T cells
