# Supplementary material for: EBV latent membrane protein 1 augments γδ T cell cytotoxicity against nasopharyngeal carcinoma by induction of butyrophilin molecules
Source: Theranostics. 2023 Jan 1;13(2):458–71. doi: 10.7150/thno.78395 (PMC9830437; doi:10.7150/thno.78395)

Table S1

Table S1. Antibodies used in this study.

Antibody against	Clone	Cat. No.	Purchased from
Western blot			
BTN2A1	polyclonal	orb499606	Biorbyt
BTN3A1	7F4F1	ABIN5684130	antibodies-online.com
p-IKKα/β	16A6	2697	Cell Signaling Technology
IKKα	3G12	19930	Cell Signaling Technology
SAPK/JNK	polyclonal	9252	Cell Signaling Technology
p-SAPK/JNK	81E11	4668	Cell Signaling Technology
STAT1	1F7C6	66545-1-lg	Proteintech
p-STAT1 (Tyr701)	58D6	88845	Cell Signaling Technology
β-Actin	8H10D10	3700T	Cell Signaling Technology
HRP donkey anti-rabbit	-	AP182P	Merck Millipore
HRP goat anti-mouse	-	AP130P	Merck Millipore
HRP goat anti-rat	-	AP131P	Merck Millipore
LMP1	CS1-4	ab78113	Abcam
GAPDH	D16H11	5174	Cell Signaling Technology
Flow cytometry			
CD3	UCHT1	558117	BD Pharmingen
Vδ2-TCR	B6	555739	BD Pharmingen
BTN2A1	polyclonal	orb484685	Biorbyt
BTN3A1	7F4F1	ABIN5684130	antibodies-online.com
PD-L1	MIH1	563741	BD Pharmingen
PD-L2	MIH18	563842	BD Pharmingen
Immunofluorescence			
Vδ2-TCR	15D	TCR1732	Thermo Fisher Scientific
BTN2A1	polyclonal	orb484685	Biorbyt
BTN3A1	7F4F1	ABIN5684130	antibodies-online.com
NLRC5	polyclonal	ab117624	Abcam
Goat anti-Mouse IgG (H+L) Cross-Adsorbed Secondary Antibody, Alexa Fluor™ 594	polyclonal	A11005	Invitrogen
Goat anti-Rabbit IgG (H+L) Highly Cross-Adsorbed Secondary Antibody, Alexa Fluor™ Plus 647	polyclonal	A32733	Invitrogen

Table S2

Table S2. Primers used for RT-qPCR.

Gene	Forward primer (5′-3′)	Reverse primer (5′-3′)
GAPDH	ACAGTCCATGCCATCACTGCC	GCCTGCTTCACCACCTTCTTG
BTN3A1	TACACAACGTCACAGCCTCT	CTCCCTTGTTGTTGCTCCAC
BTN2A1	AGGAGTACCGAGGAAGAACCA	TGTGATGTTGTGTATGACCAGG
BTN3A2	ACAGAGCGGGAAATAAGCCTA	GGACGAAGACTCCTCTCCAC
BTN3A3	CCCCGATCCTGACCTAGTG	TCTGATTGGTTGTTGCAGAAGG
BZLF1	TACAAGAATCGGGTGGCTTC	GCACATCTGCTTCAACAGGA
BRLF1	AGAGGATCAGGCCCTTCCAT	AACAGATGACTTGCCTCGGG
BRRF1	CTGCTGGAAGACACCATCGT	AGGCATGGTGTCTGTCTTGG
BXLF1	TAGAAAGGCGGCAGGTAAGC	AAGTCCGTAAACGTGGGTCC
BMRF1	AGGAGTGCTGCAGGTAAACC	GCTCTGGTGATTCTGCCACT
BARF1	GCATGCACCACGATGTCATC	TTCATGCGGCACAGGTAGTT
BCRF1	GACCCTGAAGCCAAAGACCA	TTGTTCTCACACGGCAGGAA
LMP1	GTGCGCCTAGGTTTTGAGAG	ACTGATGAACACCACCACGA
IFNG	TGAATGTCCAACGCAAGCA	CGACCTCGAAACAGCATCTGA

Table S3

Table S3. Summary of EBV genes that may influence NLRC5 expression

EBV infection phase	Viral gene category	Gene name	Viral gene function	Impact on immunity
Latent	Latent gene	LMP1	Major viral oncoprotein and maintenance of latent cell	Induces IL-1α, IL-1β, and TNF-α expression
Lytic (Productive)	IE gene	BRLF1	Lytic gene trans-activator	Reduces expression or promotes degradation of IRF3/7
		BZLF1	Lytic gene trans-activator	Increases turnover of IFN-α and IFN-γ receptors
	E gene	BXLF1	Thymidine kinase	Viral DNA replication
		BMRF1	mRNA export factor	Induces STAT1 expression
		BRRF1	Enhancer of lytic reactivation	Induces lytic protein expression
		BARF1	Macrophage colony-stimulating factor decoy receptor	Promotes cell proliferation, induces cell immortalization, and anti-apoptosis
	L gene	BCRF1	TBP-like protein important for viral late gene expression	Encodes viral IL-10 homologue, evade immune system by downregulate MHC and T cell responses

Figure S1

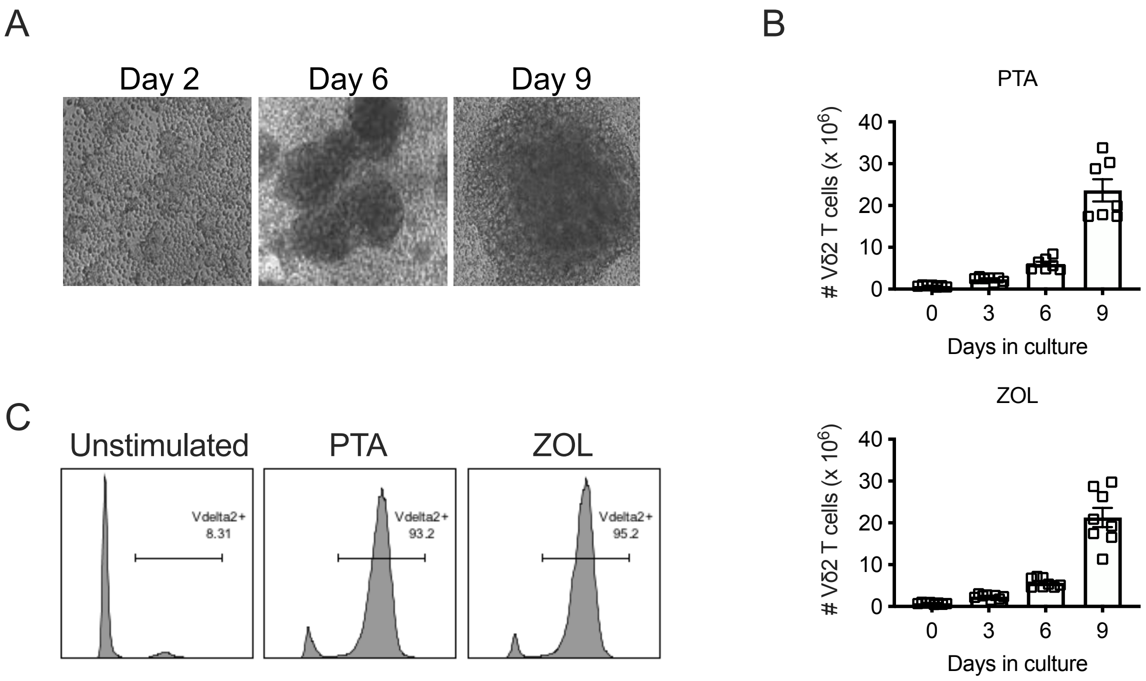

Figure S1. Expansion of Vδ2 T cells in vitro. (**A**) Detection of increasing cell clusters observed under the microscope, 100X. (**B**) Increase in the number of Vδ2 T cells over the 9-day culture. (**C**) Purity of Vδ2^{+} cells gated on CD3^{+} cells at day 9 measured by flow cytometry. Representative images and plots are shown.

Figure S2

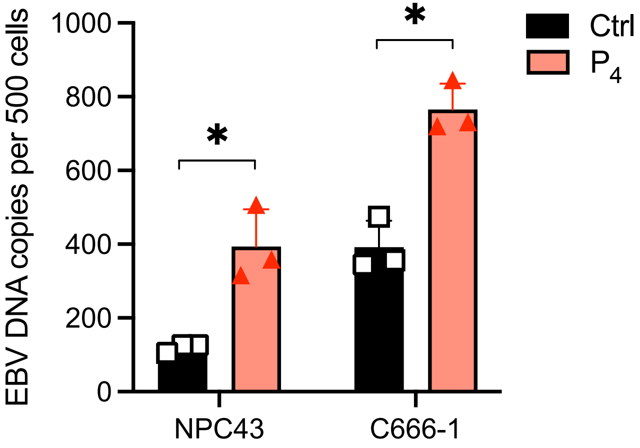

Figure S2. Induction of productive EBV infection by P_4 in EBV$^+$ NPC43 and C666-1 cell lines. Viral DNA copies of EBV was measured by qPCR using specific primers against BZLF1 and compared to a BZLF1-encoding plasmid control. Data shows mean ± SD from 3 independent experiments. Student's *t-test* was performed between Ctrl and P_4. *$P < 0.05$.

Figure S3

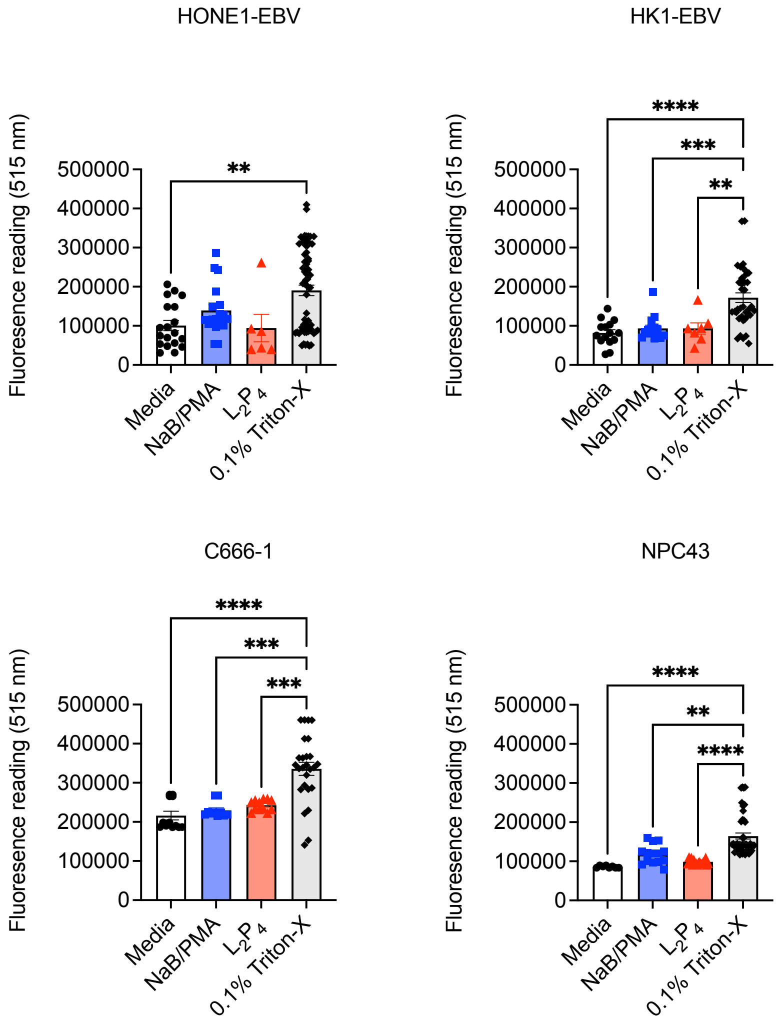

Figure S3. Induction of cell lysis by EBV-reactivating agents in NPC cells. Calcein was used to pre-label the NPC cell lines HONE1-EBV, HK1-EBV, NPC43 and C666-1 before examined for cell death induced by the EBV-reactivating agents NaB/PMA and L_2P_4, or total cell lysis with 0.1% Triton-X (as the positive control for the cytotoxicity assay in Figure 1). Cell lysis is represented by the release of the Calcein at an absorbance of 515 nm and compared to 0.1% Triton-X as positive control.

Figure S4

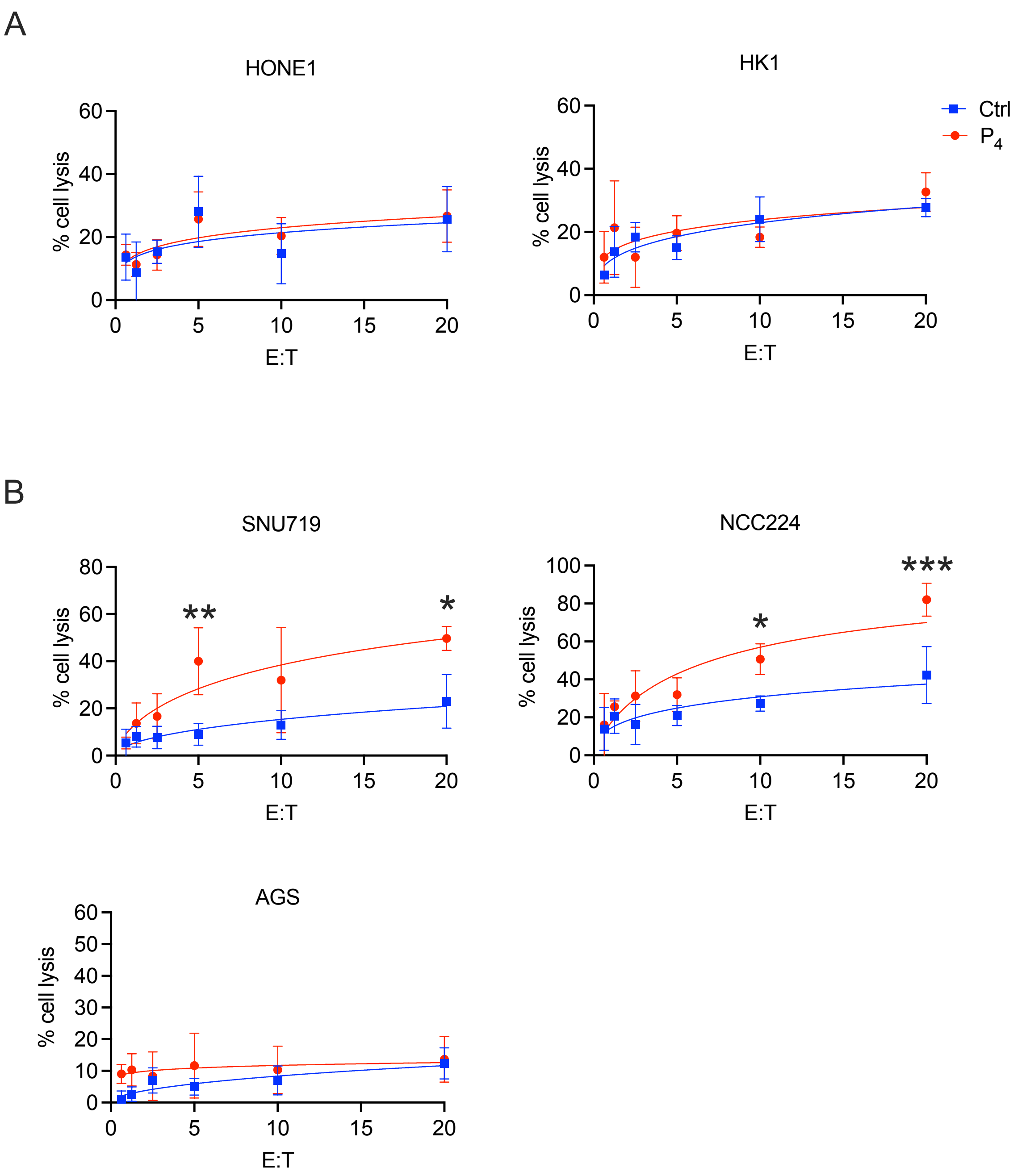

Figure S4. Vδ2 T cell cytotoxicity on tumor cell lines. Calcein cytotoxicity assay was performed on tumor cells co-cultured with PTA-expanded Vδ2 T cells at different effector: target (E:T) ratios for 4 h. Tumor cells were pre-treated with P_4 for 24 h before experiment for (**A**) EBV^- NPC cell lines (HONE1 and HK1), and (**B**) EBV^+ gastric tumor cells (SNU719 and NCC224) and EBV^- gastric tumor cell (AGS). Data represents mean ± SD from 3 independent experiments. Two-way ANOVA statistical test was performed.
$*P < 0.05$, $**P < 0.01$, $***P < 0.001$.

Figure S5

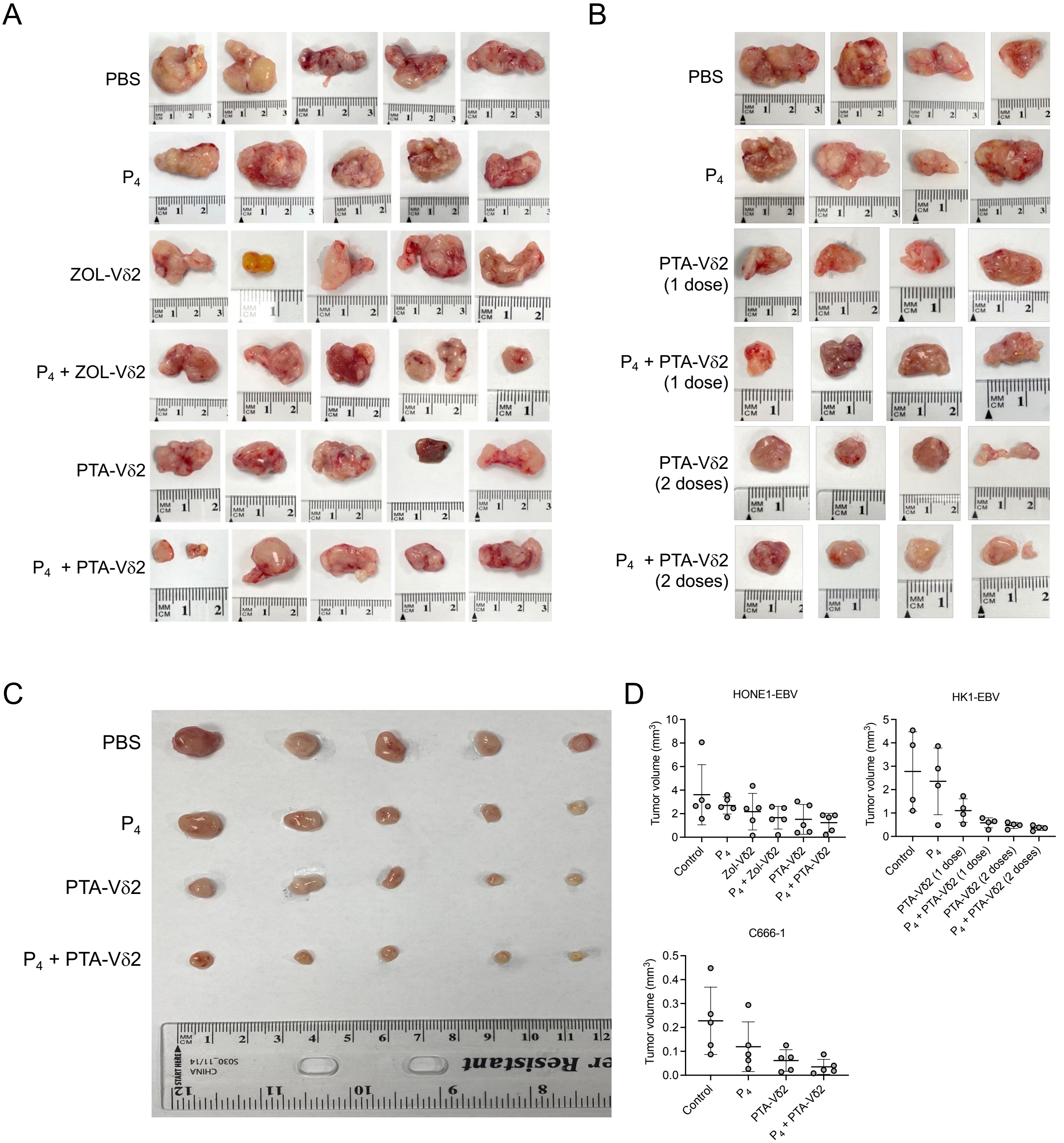

Figure S5. Photos of NPC tumors extracted from mice at experimental endpoint are shown. (**A**) HONE1-EBV, **(B)** HK1-EBV, and **(C)** C666-1 Photos were taken individually and collaged for A and B. For A, five representative photos are shown. (**D**) Column graphs showing the tumor volume (mm^3) of the photos in A, B and C.

Figure S6

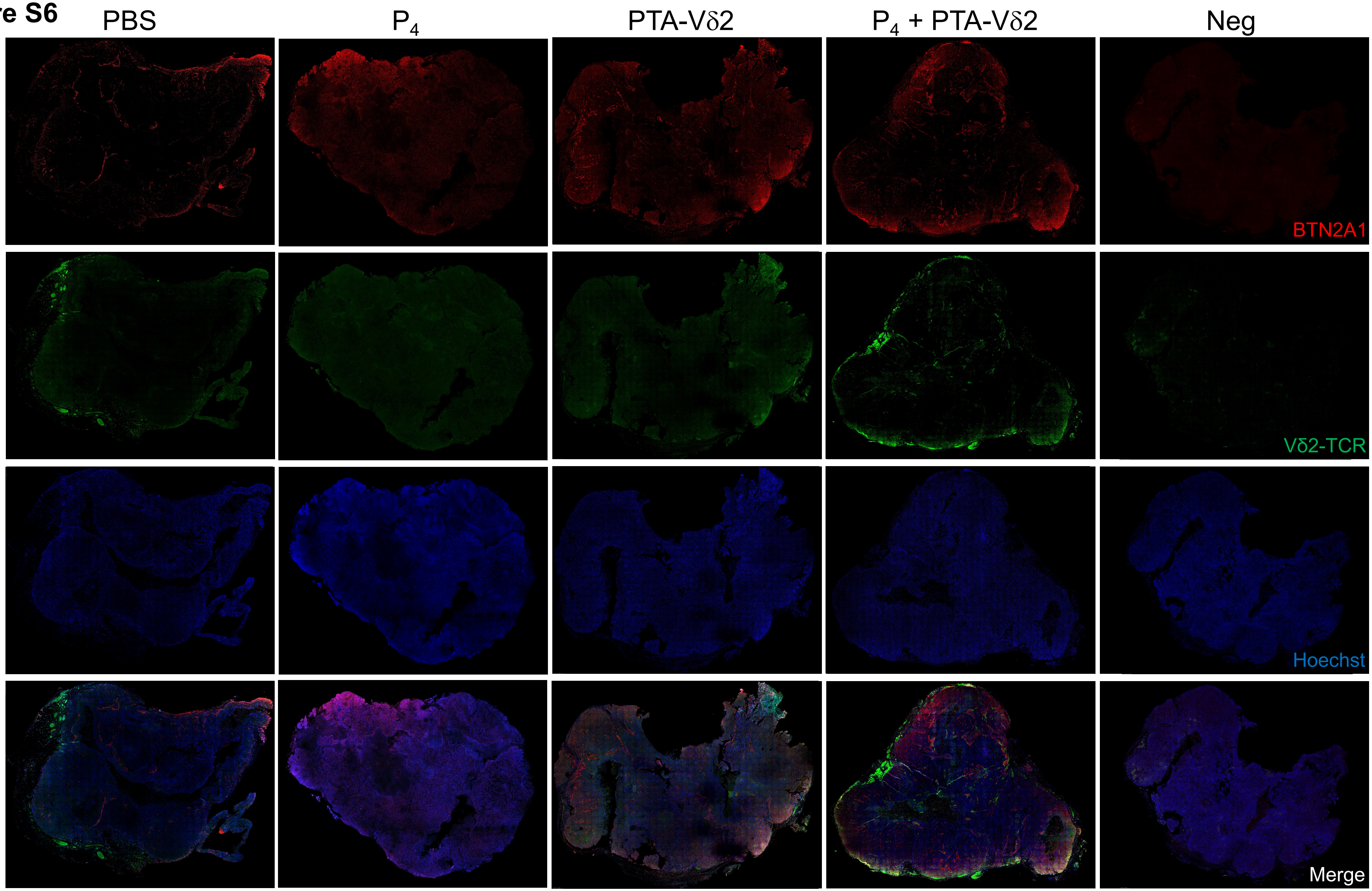

Figure S6. P_4 treatment results in increased co-localization of Vδ2 T cells and BTN2A1-expressing cells in NPC tumor in vivo. HK1-EBV tumor tissue sections from PBS, P_4, PTA-Vδ2, and P_4 + PTA-Vδ2 mice were immunostained for Vδ2-TCR$^+$ cells (green), BTN2A1 (red) and nucleus (blue) with Hoechst 33258. Representative tile scan images assessed by confocal microscopy for individual channels are shown together with Neg staining control.

Figure S7

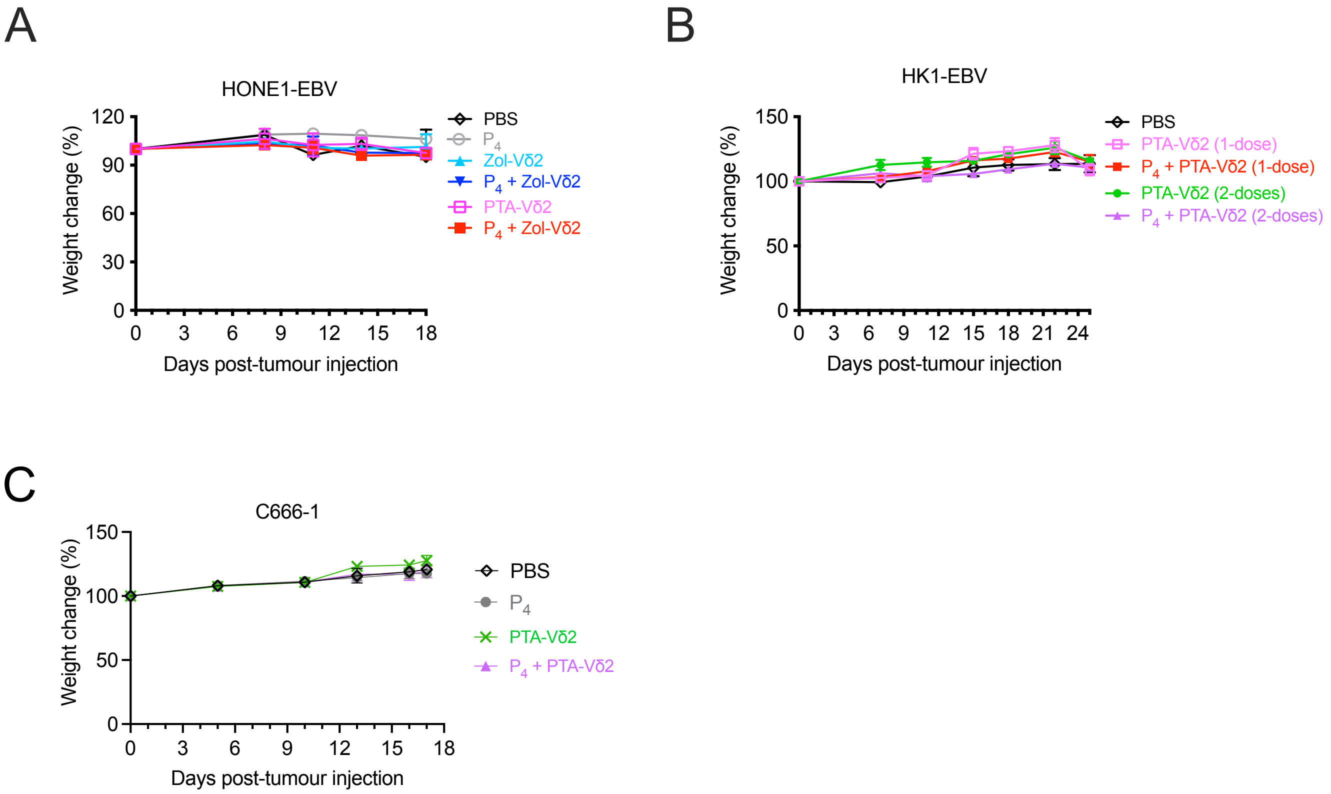

Figure S7. Weight changes of NPC-bearing NSG mice in the course of P_4 and/or Vδ2 T cell treatments. (**A**) HONE1-EBV, (**B**) HK1-EBV, and (**C**) C666-1 bearing mice in different treatment groups were measured for weight changes (% of day 0) over time.

Figure S8

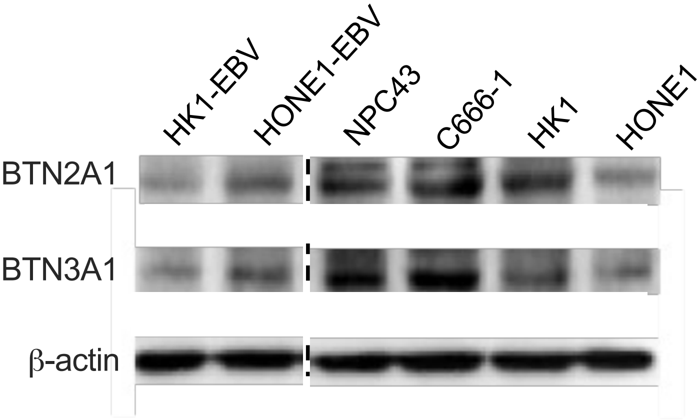

Figure S8. Western blot analysis of BTN2A1 and BTN3A1 protein expression on NPC cell lines. Representative immunoblots of BTN2A1, BTN3A1, and β-actin expression on HK1-EBV, HONE1-EBV, NPC43, C666-1, HK1 and HONE1 cells are shown.

Figure S9

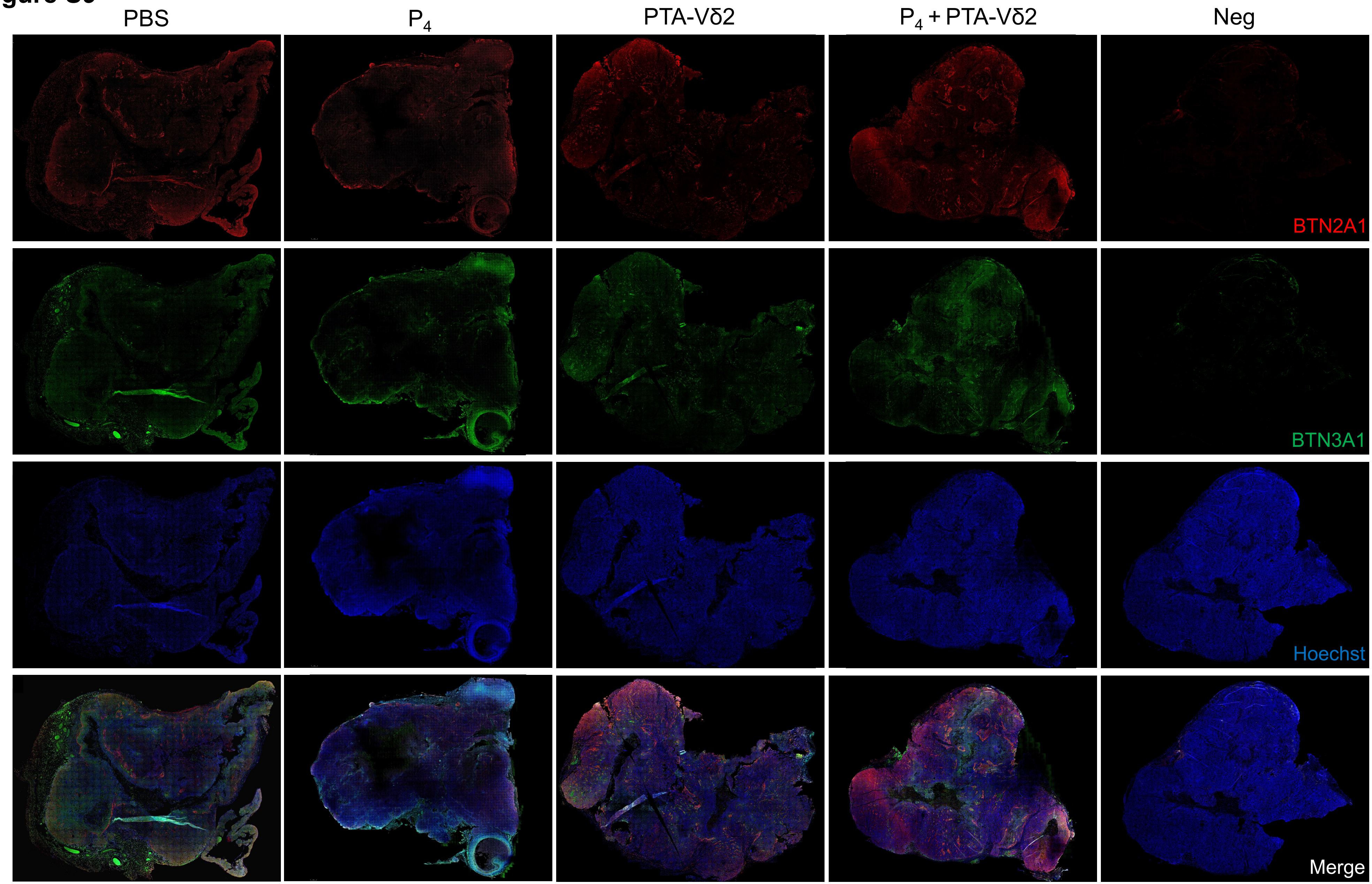

Figure S9. P4 treatment results in increased co-localization of BTN2A1 and BTN3A1-expressing cells in NPC tumor in vivo. HK1-EBV tumor tissue sections from PBS, P_4, PTA-Vδ2, and P_4 + PTA-Vδ2 mice were immunostained for BTN3A1 (green), BTN2A1 (red) and nucleus (blue) with Hoechst 33258. Representative individual tile scan images assessed by confocal microscopy are shown.

Figure S10

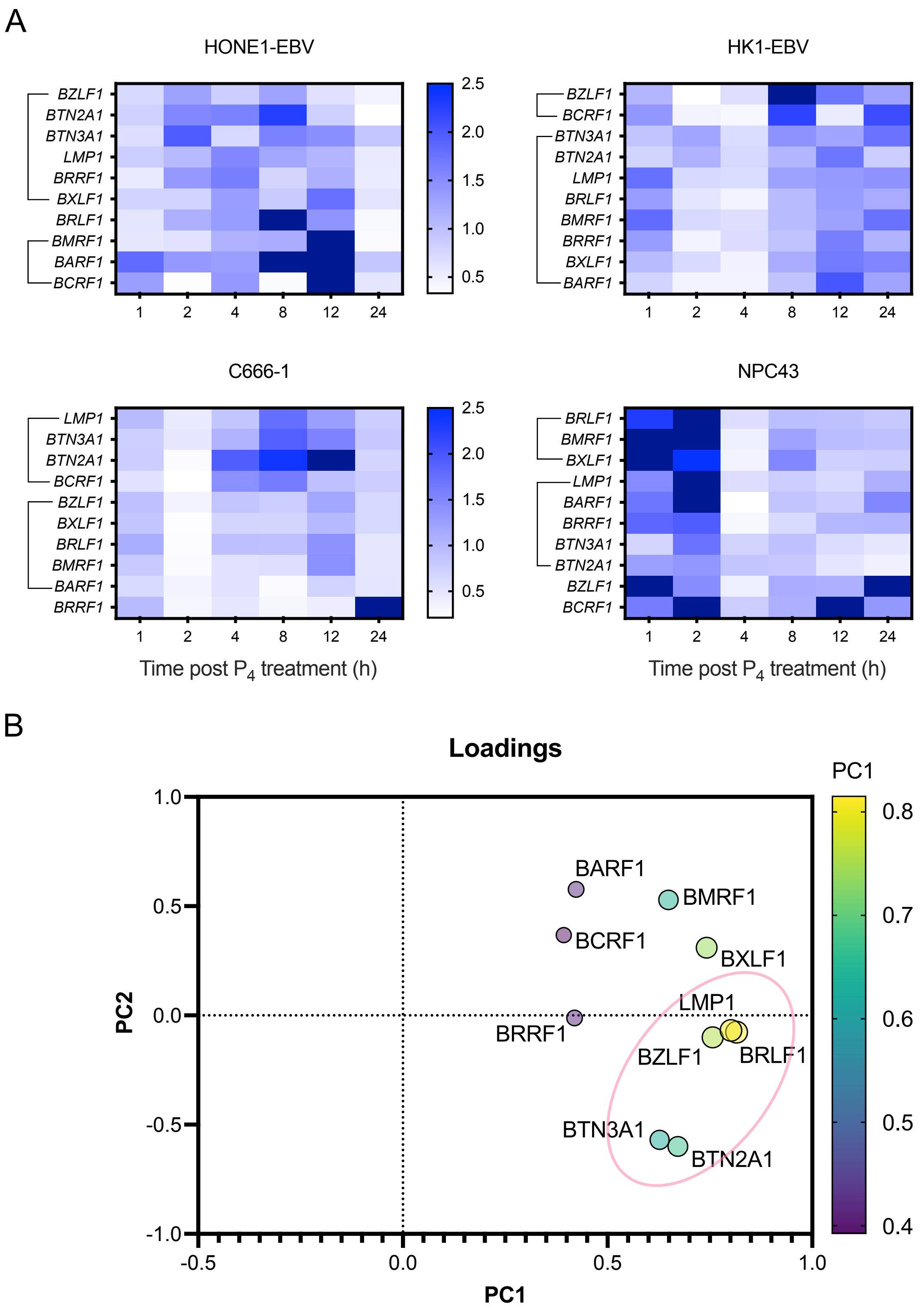

Figure S10. Analysis of EBV genes relating to butyrophilin expression. Data from RT-qPCR data of EBV genes expressed following P_4 treatment over time (1, 2, 4, 8, 12 and 24 h) were used for clustering (**A**) and principal component analysis (PCA) (**B**). (**A**) Clustering of gene expression into groups indicated by the lines for the data from the four different cell lines. (**B**) PCA analysis of the EBV genes and BTN2A1 and BTN3A1 expression to show a closer relationship between LMP1, BZLF1, and BRLF1 to the BTN genes (pink oval).

Figure S11

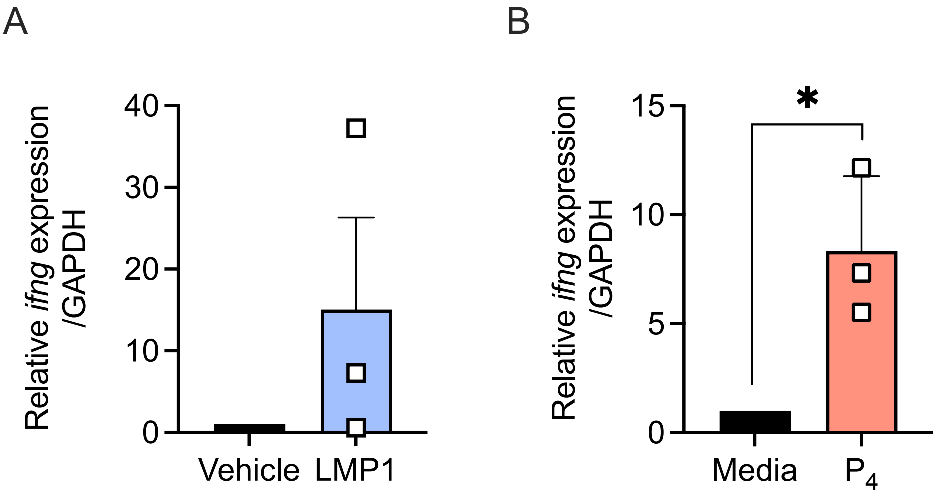

Figure S11. Induction of *ifng* expression by LMP1 or P_4. (**A**) Overexpression of LMP1 in HONE1 cells or (**B**) P_4 treatment of HONE1-EBV cells resulted in the induced expression of *ifng* measured by qRT-PCR at 3 h and 2 h post-treatment, respectively. Data represents mean ± SEM from 3 independent experiments. Student's *t*-test was performed. *$P < 0.05$.

Figure S12

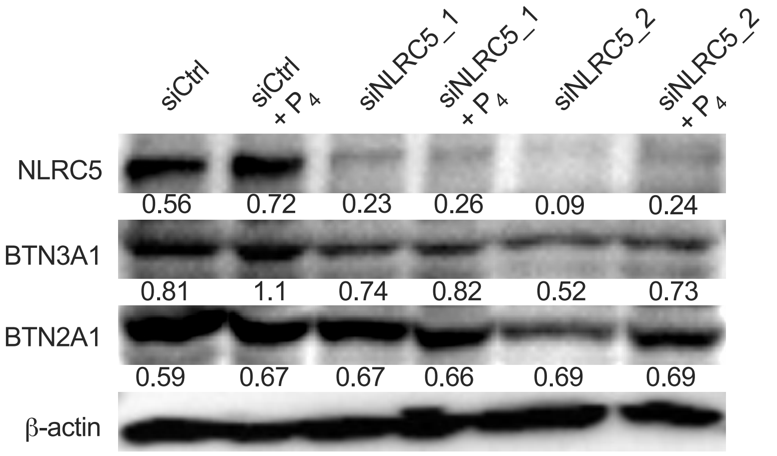

Figure S12. siRNA against NLRC5 downregulated protein expression of NLRC5, BTN3A1 and BTN2A1 in HONE1-EBV cells with or without P_4 treatment. Two siRNA against NLRC5 (siNLRC5_1 and siNLRC5_2) or scrambled control (siCtrl) were transfected into HONE1-EBV cells for 24 h before treated with P_4 for another 16 h. Protein expression of NLRC5, BTN3A1 and BTN2A1 were assessed by Western blot and the band intensity compared to β-actin control are shown as the numbers. Representative blots from three independent experiments are shown.

Figure S13 A

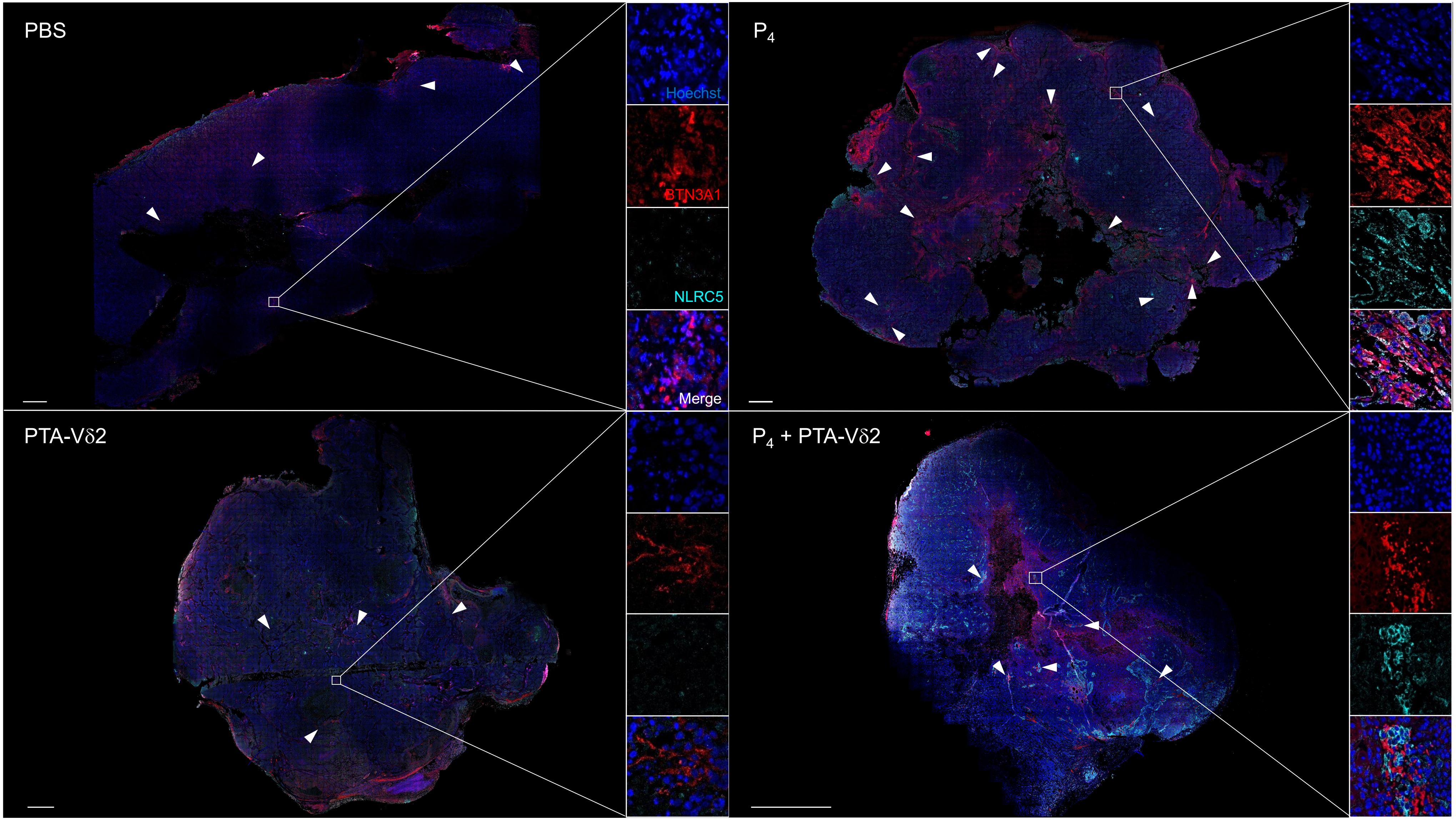

Figure S13. (A) P_4 treatment results in increased co-localization of NLRC5 and BTN3A1-expressing cells in NPC tumor in vivo. HK1-EBV tumor tissue sections from PBS, P_4, PTA-Vδ2, and P_4 + PTA-Vδ2 mice were immunostained for NLRC5 (cyan), BTN3A1 (red) and nucleus (blue) with Hoechst 33258. Scale bar represents 800 µm. Representative tile scan images assessed by confocal microscopy are shown.

Figure S13 B

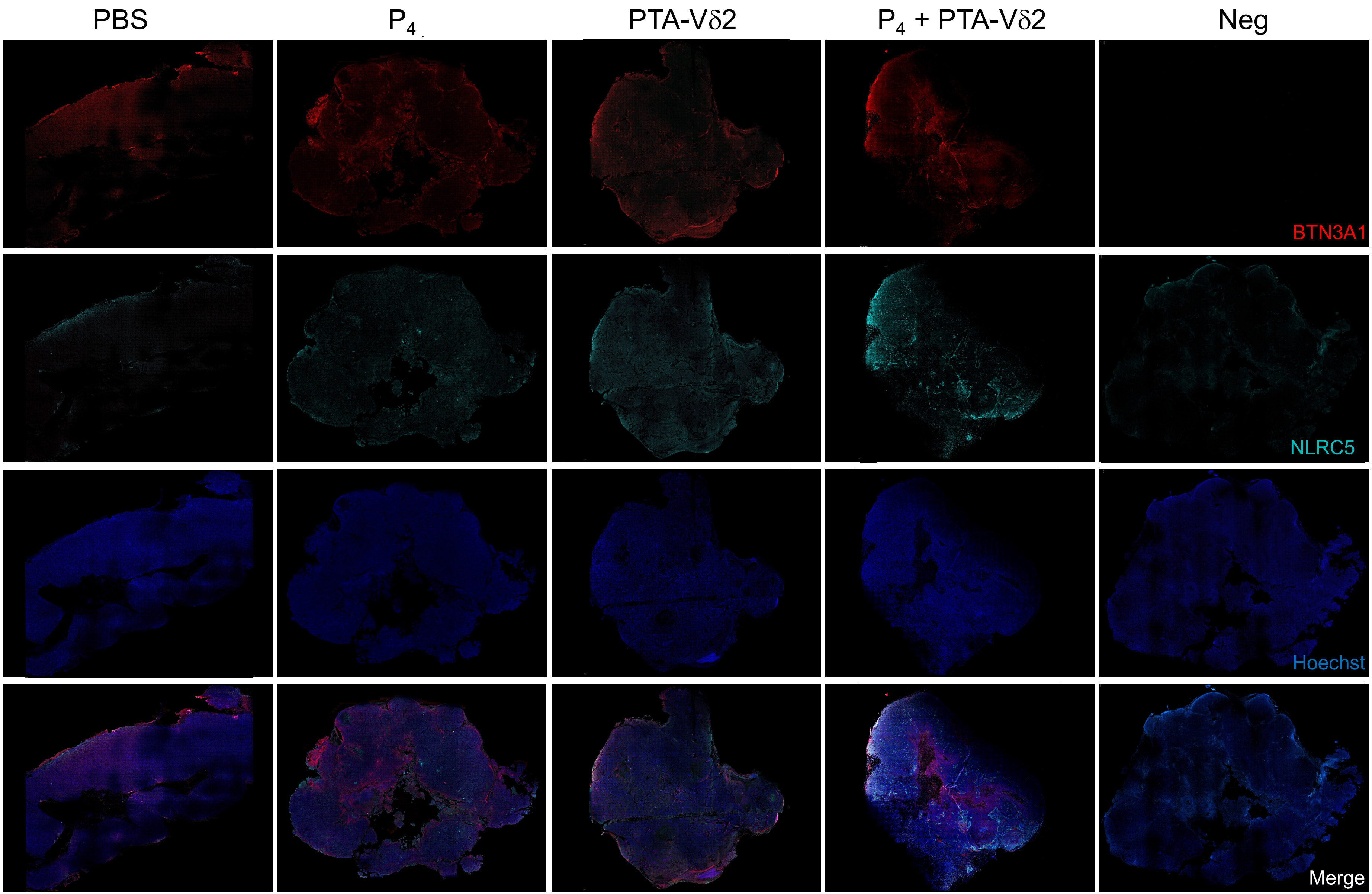

Figure S13. (B) P_4 treatment results in increased co-localization of NLRC5 and BTN3A1-expressing cells in NPC tumor in vivo. HK1- EBV tumor tissue sections from PBS, P_4, PTA-Vδ2, and P_4 + PTA-Vδ2 mice were immunostained for NLRC5 (cyan), BTN3A1 (red) and nucleus (blue) with Hoechst 33258. Representative individuals tile scan images assessed by confocal microscopy are shown.

Figure S14

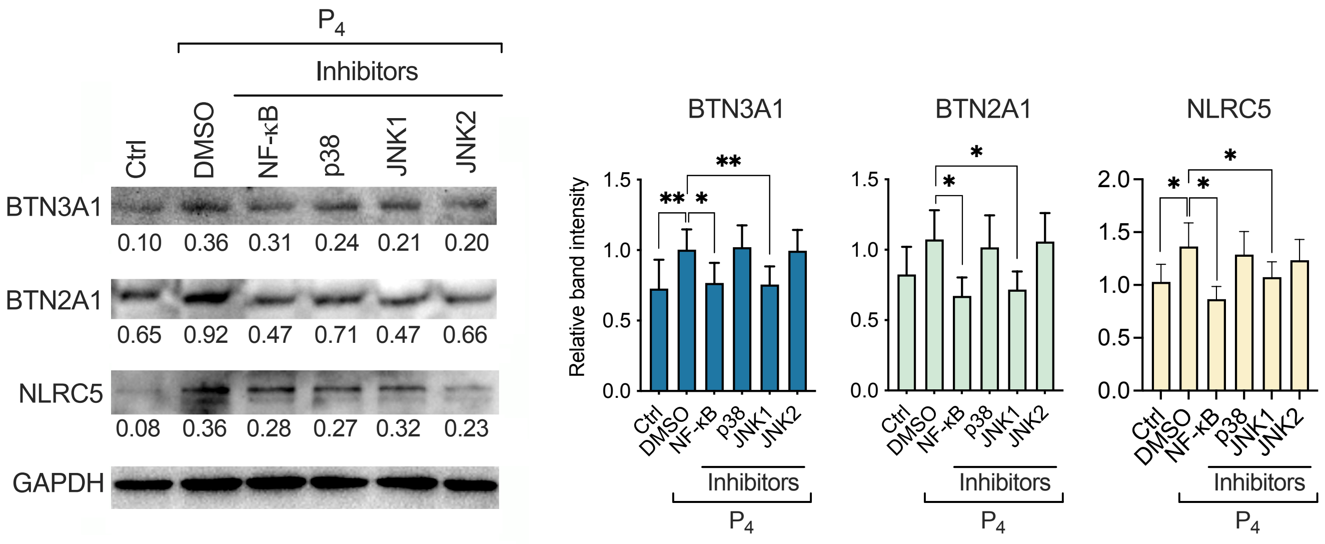

Figure S14. Western blot analysis of BTN3A1, BTN2A1 and NLRC5 expression following P_4 treatment of HONE1-EBV cells with or without inhibitors. Numbers under immunoblots indicate relative band intensity to β-actin or GAPDH adjusted for background. Data from five independent experiments are shown as mean ± SEM in the column graphs. Student's *t*-test was performed. $^*P < 0.05$, $^{**}P < 0.01$.

Figure S15

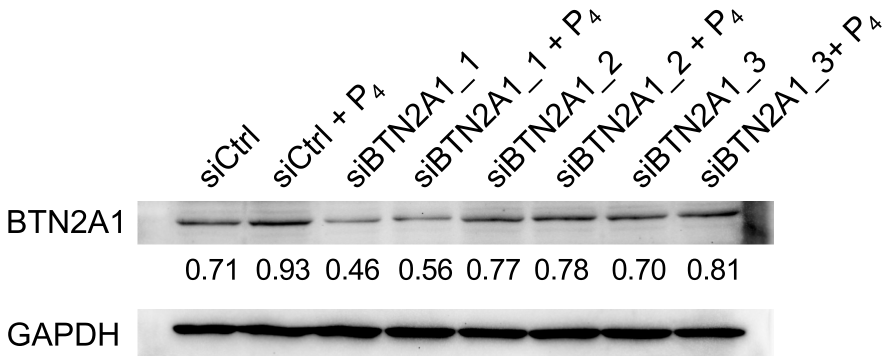

Figure S15. Effect of siRNA against BTN2A1 protein expression in C666-1 following P_4 treatment. Three siRNA against NLRC5 (siBTN2A1_1, siBTN2A1_2 and siBTN2A1_3) or scrambled control (siCtrl) were transfected into C666-1 cells for 24 h before treated with P_4 for another 16 h. Protein expression of BTN2A1 and GAPDH were examined by Western blot. Numbers represent relative band intensity based on GAPDH. siBTN2A1_1 was selected for subsequent experiments.

Original data for Western blot results

Figure 4A

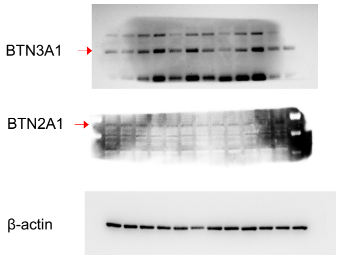

Figure 4B

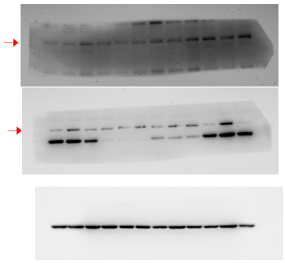

Figure 5G

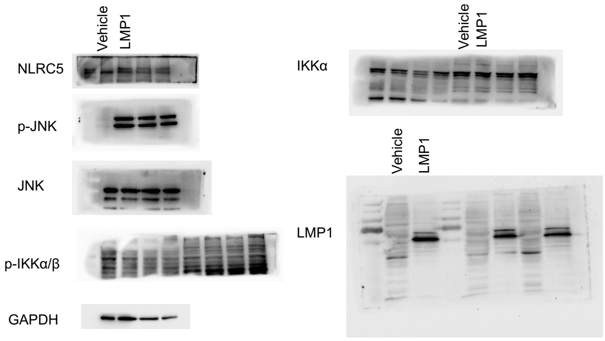

Figure 5H and Figure S15

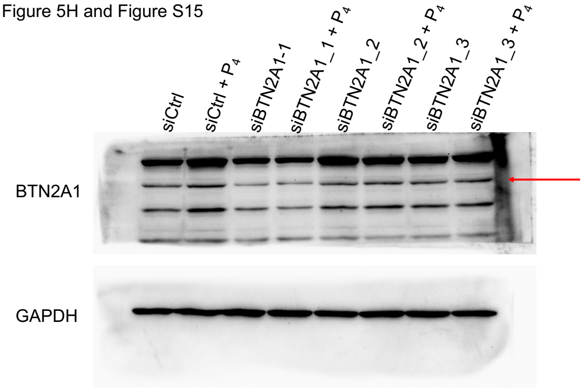

Figure S8

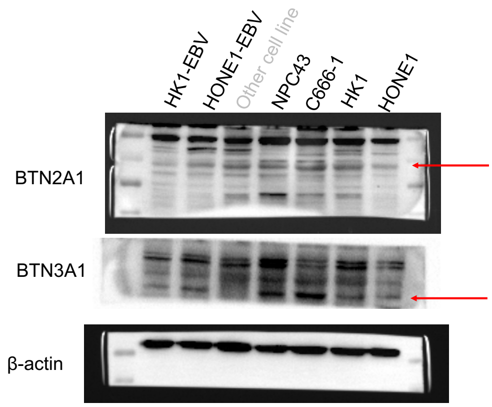

Figure S12

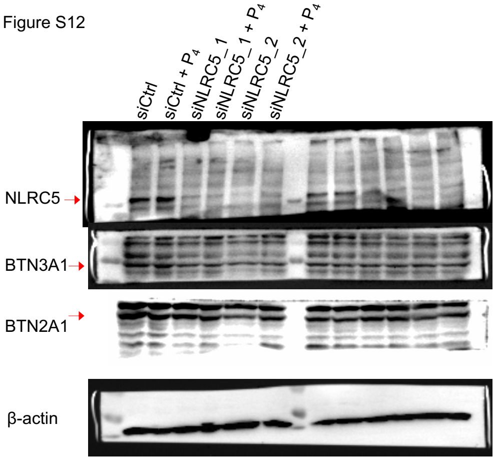

Figure S14

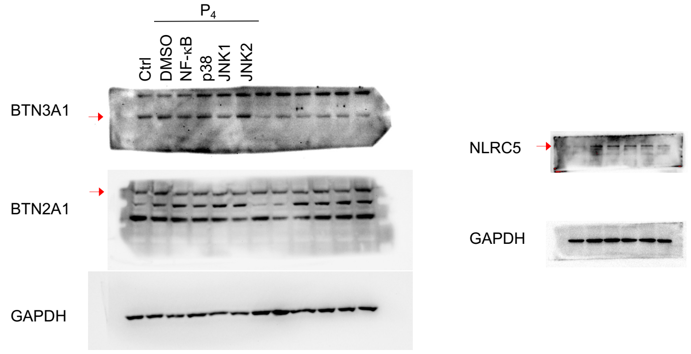

Supplement: Supplementary file 1 — Supplementary figures and tables. [file thnov13p0458s1.pdf]
